# Supplementary material for: Cognitive-behavioural therapy (CBT) for renal fatigue (BReF): a feasibility randomised-controlled trial of CBT for the management of fatigue in haemodialysis (HD) patients
Source: BMJ Open. 2018 Mar 8;8(3):e020842. doi: 10.1136/bmjopen-2017-020842 (PMC5855165; doi:10.1136/bmjopen-2017-020842)
Supplement: Author's manuscript [file bmjopen-2017-020842.draft_revisions.pdf]

BMJ Open

BMJ Open is committed to open peer review. As part of this commitment we make the peer review history of every article we publish publicly available.

When an article is published we post the peer reviewers' comments and the authors' responses online. We also post the versions of the paper that were used during peer review. These are the versions that the peer review comments apply to.

The versions of the paper that follow are the versions that were submitted during the peer review process. They are not the versions of record or the final published versions. They should not be cited or distributed as the published version of this manuscript.

BMJ Open is an open access journal and the full, final, typeset and author-corrected version of record of the manuscript is available on our site with no access controls, subscription charges or pay-per-view fees (http://bmjopen.bmj.com).

If you have any questions on BMJ Open's open peer review process please email editorial.bmjopen@bmj.com

BMJ Open

Cognitive Behavioural Therapy (CBT) for Renal Fatigue (BReF): A feasibility randomised-controlled trial of CBT for the management of fatigue in haemodialysis (HD) patients.

Journal:	*BMJ Open*
Manuscript ID	bmjopen-2017-020842
Article Type:	Protocol
Date Submitted by the Author:	29-Nov-2017

Complete List of Authors:	Picariello, Federica; King's College London, Health Psychology Section, Psychology Department<br>Moss-Morris, Rona; King's College London, Psychology Department, Insitute of Psychiatry<br>Macdougall, Iain; Kings College Hospital NHS Foundation Trust, Nephrology<br>Norton, Sam; King's College London, Psychology Department, Insitute of Psychiatry<br>Da Silva-Gane, Maria; Lister Hospital, Renal Medicine; University of Hertfordshire<br>Farrington, Ken; Lister Hospital, Renal Medicine; University of Hertfordshire<br>Clayton, Hope; Lister Hospital, Renal Medicine<br>Chilcot, Joseph; King\'s College London, Psychology

Keywords:	fatigue, Dialysis < NEPHROLOGY, Cognitive behavioural therapy, quality of life, psychotherapy

SCHOLARONE™
Manuscripts

Cognitive Behavioural Therapy (CBT) for Renal Fatigue (BReF): A feasibility randomised-controlled trial of CBT for the management of fatigue in haemodialysis (HD) patients.

v2 04.09.2017

Federica Picariello[1*], Rona Moss-Morris[1], Iain C Macdougall[2], Sam Norton[1], Maria Da Silva-Gane[3,4], Ken Farrington[3,4], Hope Clayton[3], and Joseph Chilcot[1]

[1]Health Psychology Section, Psychology Department, Institute of Psychiatry, Psychology and Neuroscience, King's College London, UK

[2]Department of Renal Medicine, King's College Hospital, London, UK

[3]Department of Renal Medicine, Lister Hospital, Stevenage, UK

[4]University of Hertfordshire, Hertfordshire, UK

* Address for Correspondence

Email: federica.picariello@kcl.ac.uk

Telephone: 02071889324

Postal address: Health Psychology Section, Psychology Department, Institute of Psychiatry, Psychology, and Neuroscience, King's College London, 5th floor Bermondsey Wing, Guy's Campus, London Bridge, London, SE1 9RT, UK

For peer review only

Trial Registration Number: ISRCTN91238019 (pre-results)

Keywords: fatigue; dialysis; cognitive behavioural therapy; kidney failure; quality of life

Word count (excluding tables and reference list): 4721

For peer review only - http://bmjopen.bmj.com/site/about/guidelines.xhtml

Abstract

Introduction: Fatigue is one of the most common and disabling symptoms in End-Stage Kidney Disease (ESKD), particularly among in-centre haemodialysis patients. This two-arm parallel group feasibility randomised-controlled trial will determine whether a fully powered efficacy trial is achievable by examining the feasibility of recruitment, acceptability and potential benefits of a CBT-based intervention for fatigue among in-centre HD patients. **Methods:** We aim to recruit 40 adult patients, undergoing in-centre haemodialysis at secondary care outpatient dialysis units, who meet clinical levels of fatigue. Patients will be randomised individually (using a 1:1 ratio) to either a 4- to 6-week CBT-based intervention (intervention arm) or to a waiting-list control (control arm). The primary feasibility outcomes include descriptive data on numbers within each recruiting centre meeting eligibility criteria, rates of recruitment, numbers retained post-randomisation, and treatment adherence. To assess the potential benefits of the BReF intervention, secondary self-report outcomes include measures of fatigue severity (Chalder Fatigue Questionnaire), fatigue-related functional impairment (Work and Social Adjustment Scale), sleep quality (Pittsburgh Sleep Quality Index), depression (Patient Health Questionnaire-9), and anxiety (Generalised Anxiety Disorder-7). Changes in fatigue perceptions (Brief Illness Perception Questionnaire), cognitive and behavioural responses to fatigue (Cognitive and Behavioural Responses to Symptoms Questionnaire), sleep hygiene behaviours (Sleep Hygiene Index), and physical activity (International Physical Activity Questionnaire –short form) will also be explored. These self-report measures will be collected at baseline and 3 months post-randomisation. Nested qualitative interviews will be conducted post-intervention to explore the acceptability of the intervention and identify any areas in need of improvement. The statistician and assessor will be blinded to treatment allocation. **Ethics and dissemination:** A National Health Service (NHS) research ethics committee approved the study. Any amendments to the protocol will be submitted to the NHS committee and study sponsor. **Trial registration:** ISRCTN91238019 (pre-results).

For peer review only - http://bmjopen.bmj.com/site/about/guidelines.xhtml

Strengths and limitations of this study

- This is the first feasibility trial assessing the feasibility, acceptability, and potential benefits of a psychological intervention for the management of fatigue among in-centre haemodialysis patients.
- The mixed-methods approach will help to evaluate comprehensively and in-depth the feasibility and acceptability of the BReF intervention; the qualitative data will complement the quantitative findings and help to identify areas in need of improvement.
- The BReF intervention was developed systematically, using theory and evidence, with substantial input from patient and public representatives.
- As this is a feasibility trial, it is not powered to detect efficacy.

For peer review only

Introduction

End-Stage Kidney Disease (ESKD) is a chronic disease of the kidneys, characterised by inadequate renal functioning, where renal replacement therapy (RRT) is necessary to sustain life [1]. Haemodialysis is the most common RRT modality, filtering toxins out of the blood via an artificial extracorporeal blood circuit. A typical HD patient will be required to attend dialysis sessions three times a week for 3-4 hours each time [2]. On average, renal patients experience 14 symptoms, with fatigue emerging as one of the most persistent and debilitating symptoms [3, 4]. Fatigue is a complex and subjective symptom characterized by extreme and persistent tiredness resistant to rest and recuperation [5-7]. Forty-nine to 92% of dialysis patients suffer from fatigue [3]. Fatigue is a substantial contributor to impaired functioning and quality of life, and recent evidence suggests that it has also implications for clinical outcomes [8-14]; yet, it is often under-recognised and under-treated by healthcare professionals, perceived as a normal consequence of the illness and treatment burden [15, 16].

Current management in the form of pharmacological treatments or exercise is ineffective [17, 18] and no theory-driven and evidence-based psychological interventions aimed at fatigue for this group currently exist, although there is some promising evidence for some improvements in fatigue following psychological interventions not aimed at fatigue specifically [19]. As the aetiology of fatigue in renal patients is still largely unknown, no consistent treatment model exists [8, 20].

There is increasing recognition regarding the importance of psychological factors in the perpetuation and maintenance of fatigue in other long-term physical conditions [21-26]. For example, in Multiple Sclerosis (MS), negative fatigue beliefs, such as catastrophizing about the consequences of fatigue, embarrassment about fatigue, and belief that fatigue is a sign of physical damage; and unhelpful behaviours in response to fatigue, like excessive resting or overdoing things followed by long resting periods to recover; were found to be strongly associated with fatigue, above and beyond the role of demographic and clinical factors, such as neurological impairment and remission status [22]. On the other hand, social support may act as a buffer to fatigue perpetuation [27]. An understanding of the contribution

For peer review only - http://bmjopen.bmj.com/site/about/guidelines.xhtml

of these factors to fatigue has translated into successful psychological interventions, leading to clinically significant improvements in fatigue severity and fatigue-related functional impairment [e.g. 28, 29]. There is, in fact, extensive evidence in support of Cognitive Behavioural Therapy (CBT) for adjustment and the management of symptoms, like fatigue, in the context of long-term physical conditions, such as cancer and MS [30, 31], despite being originally developed for the treatment of mood disorders [32, 33]. CBT is a structured, tailored and time-limited talking therapy that focuses on changing negative beliefs and unhelpful behaviours, as well as relaxation techniques, stress-management, and mindfulness to foster resilience [33, 34].

We conducted preliminary work, consisting of prospective and qualitative studies, which revealed the importance of cognitive and behavioural factors in the experience of fatigue in ESKD, in line with the findings from other long-term physical conditions. To date, the effectiveness of CBT specifically for fatigue has not been examined in this population, yet, a similar approach may also be useful here. Based on the findings from these precursor studies and Patient and Public Involvement (PPI) input, we adapted an existing CBT approach initially developed by one of the authors (RMM) for fatigue in Multiple Sclerosis. According to this renal fatigue treatment formulation, which integrates biological and psychosocial factors; there are a number of factors that may act as triggers of fatigue in this patient population, such as anaemia and haemodialysis. Whilst these factors trigger initial symptoms of fatigue, one's thoughts, emotions, and behaviours in response to fatigue may maintain and perpetuate fatigue, potentially leading to a vicious cycle of negative illness and fatigue beliefs, increased distress, and maladaptive behaviours, as displayed in *Figure 1*. The factors maintaining and perpetuating fatigue are targeted in CBT.

Objectives

A feasibility design was deemed necessary to determine whether a full-scale randomised-controlled trial (RCT) is feasible, by considering numbers meeting eligibility criteria, rates of recruitment and retention post-randomisation, floor/ceiling effects that might affect sensitivity to change; as well as by identifying any intervention-specific issues, particularly occurrence of adverse events and timing burden. The following aims will be addressed in this feasibility RCT:

- Objective 1: To estimate rates of recruitment and retention.

For peer review only

- Objective 2: To estimate willingness to be randomised.
- Objective 3: To explore the level of adherence to the intervention (intervention arm only).
- Objective 4: To estimate the standard deviation of fatigue in this patient population in order to compute a more robust estimate of the sample size required for an efficacy trial.
- Objective 5: To preliminary assess the psychometric properties of the self-report instruments used.
- Objective 6: To explore the potential benefits of the intervention at reducing fatigue severity and fatigue-related functional impairment as compared to the waiting-list control.
- Objective 7: To explore the potential benefits of the intervention at reducing depression and anxiety and improving sleep quality as compared to the waiting-list control.
- Objective 8: To examine change in fatigue-related cognitions and behaviours, and whether their effect differs between the intervention and control arm.
- Objective 9: To qualitatively explore patient perceptions of the acceptability and usefulness of the intervention and identify areas of improvement for a future full-scale trial.
- Objective 10: To explore any intervention-specific issues, particularly setting, mode of delivery of the intervention and acceptable number of sessions/chapters.

Methods

Design

A two-arm parallel group feasibility randomised-controlled trial (RCT). There will be one follow-up assessment at three months post-randomisation. A nested-qualitative study will evaluate patients' experiences with the intervention.

Setting and participants

Outpatient haemodialysis patients will be recruited from two NHS sites in England.

Inclusion and exclusion criteria

Participants are eligible for the study if they:

For peer review only - http://bmjopen.bmj.com/site/about/guidelines.xhtml

1. Are over 18 years of age,
2. Have a confirmed ESKD diagnosis
3. Are experiencing clinical levels of fatigue defined as scoring >18 on the Chalder Fatigue Questionnaire, when using the continuous scoring [35, 36].
4. Have full verbal and written proficiency in English
5. Are receiving in-centre haemodialysis
6. Length of time on dialysis > 90 days
7. Are willing and able to take part in the study and intervention

Patients will be excluded if they:

1. Do not provide informed consent or refuse to be randomised,
2. Have any known cognitive impairments
3. Have a severe mental health disorder, for example, psychosis, bipolar disorder
4. Do not have full verbal and written proficiency in English
5. Are currently receiving psychotherapy
6. Are currently participating in any other intervention trial
7. Are failing on dialysis and approaching end of life (supportive care/palliative care pathway)
8. Have a fatigue (CFQ) score below the cut-off at the pre-randomisation assessment (spontaneous improvement after screening)

Flow of recruitment and participant timeline

Recruitment will take place from October 2017 to July 2018. Patients interested in participating will be given a participant information sheet, screening questionnaire, consisting of sociodemographic and illness-related questions and the Chalder Fatigue Questionnaire; an informed consent form, and a freepost envelope. Potential participants will be given a minimum of 24 hours to establish if they would like to take part. Following consent, eligible participants will receive the baseline questionnaire, and will be randomised after the completion of the baseline questionnaire. Participants who score below the fatigue cut-off at the pre-randomization assessment will be excluded.

Participants will be informed of the outcome of the randomisation process over the phone and will receive confirmation of their treatment allocation and materials via post. We

For peer review only - http://bmjopen.bmj.com/site/about/guidelines.xhtml

anticipate the participant's journey through the study will last approximately 4-5 months, as summarised in *Figure 2*, with approximately one month dedicated to screening and randomisation. The intervention will last between 4 to 6 weeks, depending on each participant's needs. Participants are expected to complete one session per week. Follow-up data will be collected at 3-months post-randomisation (T1). On completion of the post-intervention questionnaire, a subsample of participants will be invited to take part in the qualitative interview. After completion of follow-up assessments at T1, participants in the control condition will receive the intervention manual and tasks workbook via post.

Randomisation, allocation concealment and blinding

Participants will be randomised in a 1:1 ratio to receive either the BReF intervention or to the waiting-list control. Participants will be randomised at the individual level. Randomisation will be stratified by centre and randomly varying block sizes will be used to maintain balance of numbers in each arm across the period of recruitment while maintaining allocation concealment. King's College London's Independent Randomisation Service will be used. Because the randomisation sequence is automated in real time, the allocation sequence is concealed from researchers. The trial coordinator will receive an automated email with the outcome of the randomisation procedure. The nature of the trial is such that blinding of participants cannot be achieved. Follow-up outcomes will be completed independently by participants by post or online. If baseline or follow-up questionnaires are not completed, then participants will receive reminder emails or phone calls and an assistance-based visit at the dialysis unit, by an independent researcher, who has not been involved in the intervention delivery. The statistician will remain blinded to treatment allocation.

BReF Intervention

The intervention is a tailored CBT-based self-management intervention with therapist support. The purpose of this intervention is to target individuals' fatigue beliefs and behaviours in order to facilitate coping with ReF. Further detail on the intervention can be found in *Figure 3*. Therapist guidance was deemed necessary to facilitate engagement with the programme, particularly in the formulation of a personal biopsychosocial model of fatigue and identification of unhelpful thoughts and behaviours [37-39].

For peer review only - http://bmjopen.bmj.com/site/about/guidelines.xhtml

The development of the CBT-based intervention was systematic, based on the findings of reviews and qualitative and prospective studies, with substantial input from 10 patient and public representatives and a multidisciplinary team of health psychologists, clinical psychologists, and nephrologists. The structure and content of the manual was drafted based on previous CBT interventions developed by one of our authors (RMM), such as "*Managing Your Multiple Sclerosis Fatigue: A Cognitive behavioural therapy manual*" and "*Improving Distress in Dialysis*"[40-44] and other sources [45-47].

Participants will be provided with a structured CBT manual and a tasks workbook, including goal-setting sheets. This will be accompanied by three to five sessions with either a primary researcher who has a background in Health Psychology, basic CBT training and experience in working with fatigued patient groups (FP), or a Registered Health Psychologist working in the renal setting (HC). In accordance with CBT principles, participants will be encouraged to complete tasks between sessions. Completion of these tasks has been found to be predictive of CBT outcomes [48, 49].

The manual consists of 10 chapters, accompanied by a tasks workbook for each session. Please see Table 1 for the content of each chapter and associated tasks to be completed between sessions. The programme consists of two units, the basic unit (Level 1) or the advanced unit (Level 2). In the basic unit, participants will cover 4 chapters out of the manual, three will be accompanied by therapist sessions and one will be selected according to participants' needs, established in the assessment. Participants, engaging well in the first two sessions, will be given the opportunity to cover an additional 2 chapters with the therapist over the phone (Level 2). Engagement will be discussed in clinical supervision, and will be assessed through, for example, completion of between session tasks and focus maintained during the sessions. The brief and stepped structure of the intervention was chosen to meet patients' needs, particularly their fatigability and potential concentration difficulties, according to the practical considerations previously raised with regards to the delivery of CBT in MS [50]. Sessions will be guided by participants' needs, identified through the self-monitoring tasks.

For peer review only - http://bmjopen.bmj.com/site/about/guidelines.xhtml

Table 1

Summary of the Content of the BReF Manual

Chapter	Content	Between sessions task
**Renal fatigue explained**	Understanding ReF and alternative explanations<br>A model for renal fatigue<br>Assessment of fatigue	Fatigue self-monitoring
**Finding balance in activities and rest**	Patterns of rest and activity and its effects on the body<br>Planning activity and rest<br>Exercise	Activity difficulty task<br>Activity and rest goal sheet
**Improving sleep**	Sleep hygiene<br>Maladaptive sleep patterns<br>Improving sleep	Sleep, activity, and rest goal sheet
**Learning to relax**	Diaphragmatic breathing<br>Progressive Muscle Relaxation (PMR)<br>Relaxation training: step-by-step	Relaxation diary
**Coping with emotions**	Strategies to cope with negative emotions<br>Self-assessment of negative emotions<br>Expressing emotions	Coping with negative emotions goal sheet
**Managing stress**	General tips to reduce the impact stress has on your life<br>Managing controllable and uncontrollable stressors<br>Mindfulness	Managing stress goal sheet

For peer review only - http://bmjopen.bmj.com/site/about/guidelines.xhtml

**Making use of your social support**	Creating a support network<br>Disclosure vs. keeping it to self<br>Social comparisons	Social support goal sheet
**Becoming aware of your thinking**	Common unhelpful thoughts<br>Identifying unhelpful thinking	Thought record
**Changing your thinking**	Identifying alternative thoughts	Alternative thoughts goal sheet
**Preparing for the future**	Sustaining and building upon improvements<br>Developing future goals<br>Tips for everyday life	Long-term goals worksheet

For peer review only - http://bmjopen.bmj.com/site/about/guidelines.xhtml

The first and last sessions will be face-to-face lasting one hour, while the remaining sessions will be over the phone, and will last 30 minutes. A combination of face-to-face and telephone sessions has been previously suggested for CBT in MS, to overcome possible limitations of individual delivery methods [50]. Sessions will be scheduled at times that suit the participants. Face-to-face sessions will be conducted in a private environment. For the telephone sessions, participants will be encouraged to have the sessions in a quiet and private environment and allocate sufficient time not to be rushed. Please see the TIDieR checklist for a summary (Table 2).

For peer review only

For peer review only - http://bmjopen.bmj.com/site/about/guidelines.xhtml

Table 2

TIDieR Checklist Summary

**Item 1: Brief name**	**Item 2: Rationale**	**Item 3: Materials**	**Item 4: Procedure**	**Item 5: Who provided**	**Item 6: Delivery mode**	**Item 7: Location/Setting**	**Item 8: When and how much**	**Item 9: Tailoring**	**Item 10: How well (planned)**
CBT for Renal Fatigue (BReF)	Please see pages 11-15	BReF manual and tasks workbook	Please see pages 21-24	Primary researcher who has a background in Health Psychology, and experience in working with fatigued patient groups OR Registered Health Psychologist working in the renal setting.	All sessions individual:<br>-2 sessions face-to-face<br>-1 to 3 sessions over the phone	Recruitment from outpatient haemodialysis units in the UK<br><br>Therapy sessions in a private environment	3 to 5 weekly sessions with the therapist, depending on engagement.<br><br>2 sessions face-to-face, lasting 60 minutes<br><br>1 to 3 sessions over the phone, lasting 30 minutes<br>1 session completed independently	Yes, tailored: optional session determined through the assessment in session 1. Tailored to participants' needs, identified through the self-monitoring.	Therapists will follow a structured intervention manual. Therapy sessions will be audio-recorded and assessed for fidelity by the supervisor, RMM.

For peer review only - http://bmjopen.bmj.com/site/about/guidelines.xhtml

Waiting-list control (control arm)

Participants allocated to the control arm of the study will receive their usual renal care, consisting of attending dialysis. As part of this feasibility trial, what constitutes usual care will be monitored to determine a control arm for a future efficacy trial and to handle potential contamination between the arms. After completion of the follow-up questionnaire, participants in the control group will receive the intervention manual and tasks workbook, but will not receive therapist support sessions. To minimise attrition from the control group, participants will be called to remind them that they can gain access to the program in the weeks that follow.

Clinical supervision

FP and HC will receive training on how to deliver the therapist support sessions from RMM, with approximately 3 to 4 hours of face-to-face contact, in addition to audio-recorded role-playing sessions with feedback. FP and HC will receive continuous supervision throughout the intervention from RMM, following the framework developed to support the delivery of psychological therapies with persistent physical conditions [51]. This will involve reflection and discussion of the sessions, feedback on the audio-recorded sessions and case management, particularly following the initial session with each participant in terms of the treatment plan and subsequently discussions around each participant's progress over the course of the intervention and progression to the Level 2 sessions.

Intervention fidelity

Two therapists will deliver all the intervention sessions following the detailed and structured manual developed for the patients. With permission from the participants, sought on the consent form, therapy sessions will be audio-recorded and a random sample assessed for fidelity during supervision by RMM.

Data collection and feasibility outcomes

The primary focus of this trial is the feasibility of the BReF intervention.

Primary feasibility outcomes

Feasibility will be assessed by collecting descriptive data on recruitment and retention rates and willingness to be randomised according to CONSORT feasibility and pilot trial

For peer review only - http://bmjopen.bmj.com/site/about/guidelines.xhtml

guidelines [52]. In the intervention arm, the degree of adherence to the intervention will also be assessed by recording completion of the chapter selected in session 1 and completed by participants independently in week 3, and the between sessions tasks, as well as recording of attendance at therapist sessions (i.e. did not attend (DNA's) and adherence to the assigned session time). Uptake of and adherence to the Level 2 sessions will also be recorded. Given the exploratory nature of this trial, the number of completed intervention components will be assessed, this may help to identify an adherence cut-off for a future efficacy trial.

Secondary self-reported patient outcomes

Self-reported patient outcomes will also be collected at baseline (T0) and 3-months post-randomisation (T1) via post or online. The assessment schedule completed by patients is summarised in Table 3 and the self-report instruments used are described below:

<u>Fatigue severity</u>

Chalder Fatigue Questionnaire (CFQ)[35]. This instrument measures fatigue severity via 11-items scored against a four-point Likert-type response scale. Scores are assigned for each response, using continuous scoring from 0 to 3. A cut-off of greater than 18 defines a fatigue case [35, 36]. Higher scores represent greater fatigue severity. The total score will be used here following recent psychometric evidence [53, 54]. This scale displays excellent psychometric properties [35, 55] and has been validated among HD patients [54].

<u>Fatigue-related functional impairment</u>

Work and Social Adjustment Scale (WSAS)[56]. The scale consists of five items that correspond to impairment in work, home management, social activities, private leisure activities and relationships as consequence of an illness or symptom, in this case fatigue. Higher scores indicate greater impairment. It has good psychometric properties [56] and has been previously used with HD patients [57].

<u>Depression</u>

Patient Health Questionnaire-9 (PHQ-9)[58]. This instrument measures depression over the last two weeks via a 9-items scale and an additional item to assess the impact of depression on functioning. Each item is scored from 0 (not at all) to 3 (nearly every day), with greater

For peer review only - http://bmjopen.bmj.com/site/about/guidelines.xhtml

scores representing greater severity of depression. Depression severity cut-offs are available. The functional item is rated from "Not at all difficult" to "Extremely difficult". The PHQ-9 displays excellent psychometric properties and is responsive to change [59, 60]. The PHQ-9 has been validated in haemodialysis [61].

Anxiety

Generalised Anxiety Disorder-7 (GAD-7)[62]. This instrument measures anxiety over two weeks, via a 7-items scale, and an additional item to assess the impact of anxiety on functioning. Each item is scored from 0 (not at all) to 3 (nearly every day), with greater scores representing greater severity of anxiety. Anxiety severity cut-offs are available. The functional item is rated from "Not at all difficult" to "Extremely difficult". The PHQ-9 displays excellent psychometric properties [62, 63]. This instrument has been used across chronic conditions, including haemodialysis patients [e.g. 40].

Sleep quality

Pittsburgh Sleep Quality Index (PSQI)[64]. This instrument measures 7 components of sleep quality (subjective sleep quality, sleep latency, sleep duration, sleep efficiency, sleep disturbance, use of sleep medication, daytime dysfunction) over a one-month time interval, via 19-items. Items are scored on an interval scale from 0 to 3. The scores of the components are then summed to obtain a global sleep quality score, ranging from 0 to 21. Higher scores indicate worse sleep quality. This scale displays satisfactory psychometric quality across patient populations [64, 65] and is widely used and has been validated [66].

Process variables

Fatigue perceptions

Brief Illness Perceptions Questionnaire (BIPQ) [67]. This instrument relies on a single-item approach to measure fatigue perceptions. It is a shorter version of the original Illness Perception Questionnaire (IPQ)[68], with moderate to good associations between the two [67]. Five of the items assess cognitive illness/symptom representations (consequences, timeline, personal control, treatment control and identity), two of them assess emotional representation (concern and emotions) and one item assesses illness/symptom comprehension. The items are rated using a response scale of 0 to 10. The psychometric properties of this measure have been assessed using samples from several illness groups, including renal disease [67], displaying satisfactory quality.

For peer review only - http://bmjopen.bmj.com/site/about/guidelines.xhtml

Cognitive and behavioural responses to fatigue

Cognitive and Behavioural Responses to Symptoms Questionnaire (CBSQ) [69]. The scale includes five cognitive subscales; fear avoidance, embarrassment avoidance, catastrophizing about symptoms, beliefs that symptoms signal damage to the body (damage beliefs), and symptom focus. There are also two behavioural subscales; resting and avoidance of activity and all-or-nothing behaviour. All items are scored on a five-point frequency scale ranging from never (0) to all the time (4). Item scores are added from each subscale to obtain a total score. Across studies, this instrument displays acceptable psychometric quality [70] and it has been used with different patient populations, including HD patients [57].

Sleep hygiene behaviours

Sleep Hygiene Index (SHI)[71]. This instrument measures sleep-related behaviours via 13 items. Each item is rated on a five-point scale, ranging from 0 (never) to 4 (always). Total scores range from 0 to 52, with higher scores representing poorer sleep hygiene. This scale displays adequate reliability and validity [71, 72]. However, it has not yet been validated in kidney failure.

Physical activity

International Physical Activity Questionnaire –short form (IPAQ-SF)[73]. This instrument measures self-reported weekly time spent on physical activities (walks, physical exertion of moderate and vigorous intensities) and inactivity (sitting) via 7 items. The questionnaire can be scored categorically according to developed cut-offs to classify individuals into low, moderate, or high physical activity groups; or it can be scored continuously. Responses can be converted to Metabolic Equivalent Task minutes per week (METmin/wk), according to the IPAQ scoring protocol. MET scores across the three sub-components can be summed to indicate overall physical activity [73]. The IPAQ is the most widely validated questionnaire, however, with some inconsistent evidence on its reliability and validity [74]. Given its brevity, simplicity, and extensive use across research, it was selected here to measure physical activity.

Adverse events

Information about occurrence of serious adverse events since the start of the study will be collected by self-report post-intervention, according to good clinical practice

For peer review only - http://bmjopen.bmj.com/site/about/guidelines.xhtml

guidelines. Adverse events will be flagged up to the trial management team and participants will be contacted to further assess the adverse event and its relationship to the study.

Other treatments

Participants will be asked whether they have received any pharmacological, psychological, or exercise-based treatment for depression and/or anxiety and/or fatigue in addition to BReF since starting the study.

Table 3

Schedule of Assessments

	TIME		
**Assessment**	Screening	Baseline (T0)	Post-intervention (T1)
**CFQ**	x	x	x
**WSAS**		x	x
**PHQ-9**		x	x
**GAD-7**		x	x
**PSQI**		x	x
**BIPQ**		x	x
**CBSQ**		x	x
**SHI**		x	x
**IPAQ-short**		x	x
**CCI**		x	
**Sociodemographic characteristics**	x	x	
**Clinical characteristics**	x	x	
**Biochemical outcomes**		x	x
**Self-reported adverse events**			x
**Self-reported treatments for distress or fatigue during study**			x
**Qualitative interviews**			x

For peer review only - http://bmjopen.bmj.com/site/about/guidelines.xhtml

Demographic, social-situational and clinical characteristics

At baseline, socio-demographic characteristics, including: gender, age, ethnicity, marital status, employment status, education, living arrangements, exercise, smoking status, and alcohol consumption; and clinical characteristics, including: dialysis vintage and receipt of anaemia treatments will be collected via self-report.

Extra renal comorbidity will be assessed at baseline by consultant nephrologists using the Charlson Comorbidity Index (CCI) [75]. This instrument is a weighted index that takes into account the number and the seriousness of comorbid diseases and is adjusted for age. The method of classifying comorbidity provides a simple, readily applicable and valid method of estimating risk of death from comorbid disease for use in longitudinal studies [75]. The CCI has been previously used with and determined suitable for dialysis patients [76, 77].

Clinical information, including: dialysis adequacy (Urea Reduction Ratio), inter-dialytic weight gain, haemoglobin, ferritin, serum albumin, creatinine, urea, phosphate, potassium, calcium, CRP, and primary renal diagnosis will be extracted from patients' medical notes at baseline (T0) and post-intervention (T1).

Qualitative interviews

To complement the quantitative process evaluation and further explore the acceptability of the intervention, in line with current MRC process evaluation guidelines [78], qualitative interviews will be conducted with a subgroup of participants from the intervention group at 3-months post-randomization (T1). The interviews will be semi-structured and will be conducted over the phone or face-to-face, in a private environment. The interviews will be conducted by an independent researcher, who has not been involved in the intervention delivery. The main aim of the interviews will be to gather participants' experiences of the intervention, to identify areas of improvement. Purposive maximum variation sampling will be employed to ensure variability of the sample across a range of sociodemographic and clinical characteristics [79], in particular: age, gender, ethnicity, dialysis vintage, degree of adherence to the intervention, degree of improvements in outcomes following the intervention. A minimum of 10 interviews will be conducted, until data saturation is reached, meaning the point where no new data is obtained with every new interview [80].

For peer review only - http://bmjopen.bmj.com/site/about/guidelines.xhtml

Sample size

The renal service of KCH has approximately 550 HD patients and Lister Hospital has approximately 510 HD patients, in which we expect to be able to approach 636 (60%) during the recruitment period. Past psychological research in dialysis patients, conducted by the team, suggest consent rates between 50-70%, assuming a more conservative uptake of 40%, 254 patients are expected to be screened, with approximately 30% (N=76) expected to meet the inclusion criteria, including around half reporting clinical levels of fatigue [57]. Assuming 50% of those eligible will consent to be randomised (N=38), a sample size of 40 participants would allow us to estimate the true population consent rate with a 11% margin of error (95% binomial exact confidence level) for those meeting eligibility criteria. In line with recommended sample sizes of pilot feasibility trials [81-83], 40 patients is deemed sufficient to explore feasibility, acceptability, and potentially efficacy of the intervention, assuming retention rates of 80%, the true population consent rate will be with a margin of error of 13% (95% binomial exact confidence interval) – an acceptable level of error, based on the time, budget and workforce constraints, as FP will act as both the trial coordinator and therapist.

Analysis plan

Descriptive statistics of patients approached, screened, eligible, consented, and randomised will be computed to address Objectives 1-2. Reasons for non-consent, exclusion, and drop-out, at each stage of the study, will be recorded and reported. Adherence to the intervention will be reported using descriptive statistics to address Objective 3. The following values will be computed: mean number of homework tasks completed, mean number of sessions completed, a breakdown on the number of participants completing each session, number of participants completing the independent chapter, mean duration of the telephone and face-to-face sessions. Standard deviations by trial arm will be computed for the fatigue outcomes in order to estimate a more robust sample size for a future efficacy trial, thereby, addressing Objective 4.

The psychometric quality of the self-report instruments used will be assessed to address Objective 5. Reliability will be assessed using Cronbach's alpha, with a minimum acceptable cut-off at α= 0.70, but preferably at α= 0.80 or higher, particularly for the key variables [84] and individual items will be checked to ensure that there are no problematic items for this patient population. Convergent validity will be assessed via Pearson's correlations between psychological constructs (e.g. depression and fatigue severity) and

For peer review only - http://bmjopen.bmj.com/site/about/guidelines.xhtml

clinical markers (e.g. comorbidities and fatigue severity). Additionally, content validity of the fatigue measures will be considered based on the qualitative data to ensure that the selected measures capture changes described by participants. Responsiveness/sensitivity to change will be assessed by checking correlations between change scores on key variables of interest and by triangulation with patients' narratives.

Given the feasibility nature of the trial, statistical significance will not be assessed; instead effect sizes and confidence intervals will be estimated. An ANCOVA will be performed to estimate the post-intervention mean difference in outcomes, controlling for the baseline levels of each outcome, for the following variables: fatigue severity, fatigue-related functional impairment, depression, anxiety and subjective sleep quality, thereby addressing Objectives 6 and 7. Group allocation will be included as an indicator variable following the intention-to-treat principle. Recruitment centre will also be controlled for in the analysis as it is a stratification factor. Differences in intervention effects by sociodemographic and clinical factors on fatigue outcomes will be explored.

Changes in fatigue perceptions, and cognitions and behaviours in response to fatigue will be evaluated via ANCOVAs to address Objective 8. The proportion of the treatment effect that may be accounted for by these process variables with confidence intervals will also be calculated, as there will be insufficient power for mediation analyses.

To meet Objective 9 and qualitatively explore the acceptability and usefulness of the intervention from the perspective of the participants, the semi-structured qualitative interviews will be transcribed verbatim and analysed using inductive thematic analysis with the use of *NVivo* software. Thematic analysis revolves around identifying recurrent themes and patterns from the interviews and developing a coding manual [85].

To address Objective 10, a mixed methods approach will be used, drawing on both the quantitative and qualitative findings to determine any intervention-specific issues, such as the optimal number of sessions.

Considering issues relating to recruitment and retention rates, suitability of the selected measures, as well as any intervention-specific issues, will help us to determine whether to proceed to a full-scale efficacy trial, if so, these findings will also inform aspects

For peer review only - http://bmjopen.bmj.com/site/about/guidelines.xhtml

of the design of the efficacy trial, such as the required sample size and appropriate self-report measures to ensure sufficient power and sensitivity to detect any intervention effects.

Discussion

Fatigue is common in chronic haemodialysis (HD) patients with consequences on patients' functioning and daily living, as well as implications on clinical outcomes. BReF is a theory-driven and evidence-based CBT intervention with therapist support aimed at improving renal fatigue, that has been designed following the MRC guidance for developing and evaluating complex interventions [86].

This is the first feasibility RCT to examine whether a fatigue-specific CBT-based programme with therapist support is feasible, acceptable, and possibly beneficial at reducing fatigue severity and fatigue-related functional impairment in fatigued patients undergoing in-centre HD. The brief and stepped structure of the intervention may be more appropriate for patients receiving in-centre HD. Prior to proceeding to a full-scale trial, it is important to identify unique challenges with recruitment and retention in this particular setting and to explore whether the content and structure of the manual are deemed useful and relevant by patients. The results of the BReF trial will inform the design of a future full-scale trial powered to detect the efficacy of CBT for the management of fatigue in HD, accompanied by a longer follow-up to assess any sustained effects of the intervention on outcomes.

Ethics

The trial received ethical approval from the London Bridge NHS Research Ethics Committee (17/LO/1406) and is co-sponsored by King's College London and King's College Hospital NHS Foundation Trust. Patient consent will be obtained. The Chief Investigator (CI) and all members of the research team will preserve the confidentiality of participants taking part in the study and will work in accordance with the Caldicott Principles, Data Protection Act 1998, NHS Code of Confidentiality and any relevant NHS Trust organisational policies. All serious adverse events related to the study will be reported to the study sponsor, ethics committee and relevant NHS R&D departments. Authorisation will be sought from the study sponsor for any future substantial and non-substantial amendments arising during the course of the study, prior to submission to the HRA. The study may be subject to inspection and

For peer review only - http://bmjopen.bmj.com/site/about/guidelines.xhtml

audit by King's College London under their remit as sponsor and other regulatory bodies to ensure adherence to GCP and the NHS Research Governance Framework for Health and Social Care (2nd edition).

Indemnity

The study is co-sponsored by King's College London and King's College Hospital, providing insurance for the study, through its own professional indemnity for research involving human participants and no fault compensation and the Trust having a duty of care to patients via NHS indemnity cover, in respect of any claims arising as a result of clinical negligence by its employees, brought by or on behalf of a study patient.

Dissemination

We will endeavour to publish the findings of this trial in a peer-reviewed journal and present the findings at relevant national and international conferences.

Contributors

All authors (FP, RMM, ICM, SN, MDS-G, KF, HP, JC) contributed to the design of the study and writing of the protocol. FP, RMM, JC, ICM were involved in the development of the BReF intervention. FP, JC, and SN were involved in the statistical analysis plan.

The day-to-day management of the study will be co-ordinated by the CI and the trial management team. The team will also meet regularly, once a month, to discuss the overall running of the study, including: rates of recruitment, adherence to the protocol, and safety of patients.

Trial Status

The study will start recruitment at the end of October 2017. Recruitment will continue until July 2018. Patient involvement in the study will conclude in November 2018.

Funding

This work is embedded within a larger PhD project funded by a Biomedical Research Studentship to Miss Federica Picariello from the National Institute for Health Research (NIHR) Biomedical Research Centre at South London and Maudsley NHS Foundation Trust

For peer review only - http://bmjopen.bmj.com/site/about/guidelines.xhtml

For peer review only

and King's College London. The views expressed are those of the authorsand not necessarily those of the NHS, the NIHR or the Department of Health.

Conflict of interest

We hereby declare that to our knowledge there are no conflicts of interest. This work has not been published previously in whole or part.

For peer review only

References

1. Haynes, R.J. and C.G. Winearls, *Chronic kidney disease.* Surgery (Oxford), 2010. **28**(11): p. 525-529.
2. Mactier, R., N. Hoenich, and C. Breen, *Haemodialysis.* , 2009, The Renal Association.
3. Almutary, H., A. Bonner, and C. Douglas, *Symptom burden in chronic kidney disease: A review of recent literature.* Journal of Renal Care, 2013. **39**(3): p. 140-150.
4. Murtagh, F.E., et al., *Symptoms in advanced renal disease: a cross-sectional survey of symptom prevalence in stage 5 chronic kidney disease managed without dialysis.* Journal of Palliative Medicine, 2007. **10**(6): p. 1266-1276.
5. Cella, D., et al., *Progress toward guidelines for the management of fatigue.* Oncology (Williston Park, NY), 1998. **12**(11A): p. 369-377.
6. Dittner, A.J., S.C. Wessely, and R.G. Brown, *The assessment of fatigue: a practical guide for clinicians and researchers.* Journal of psychosomatic research, 2004. **56**(2): p. 157-170.
7. Ream, E. and A. Richardson, *Fatigue: a concept analysis.* International journal of nursing studies, 1996. **33**(5): p. 519-529.
8. Artom, M., et al., *Fatigue in advanced kidney disease.* Kidney International, 2014. **86**(3): p. 497-505.
9. Jhamb, M., et al., *Correlates and outcomes of fatigue among incident dialysis patients.* Clinical Journal of the American Society of Nephrology, 2009. **4**(11): p. 1779-1786.
10. Jhamb, M., et al., *Impact of fatigue on outcomes in the hemodialysis (HEMO) study.* American Journal of Nephrology, 2011. **33**(6): p. 515-523.
11. Koyama, H., et al., *Fatigue is a predictor for cardiovascular outcomes in patients undergoing hemodialysis.* Clinical Journal of the American Society of Nephrology, 2010. **5**(4): p. 659-666.
12. Davison, S.N. and G.S. Jhangri, *Impact of pain and symptom burden on the health-related quality of life of hemodialysis patients.* Journal of Pain and Symptom Management, 2010. **39**(3): p. 477-485.
13. Bonner, A., M. Caltabiano, and L. Berlund, *Quality of life, fatigue, and activity in Australians with chronic kidney disease: A longitudinal study.* Nursing & Health Sciences, 2013. **15**(3): p. 360-367.
14. Bossola, M., et al., *Fatigue Is Associated with Increased Risk of Mortality in Patients on Chronic Hemodialysis.* Nephron, 2015. **130**(2): p. 113-118.
15. Weisbord, S.D., et al., *Renal provider recognition of symptoms in patients on maintenance hemodialysis.* Clinical Journal of the American Society of Nephrology, 2007. **2**(5): p. 960-967.

For peer review only - http://bmjopen.bmj.com/site/about/guidelines.xhtml

16. Lee, B.O., et al., *The fatigue experience of haemodialysis patients in Taiwan.* Journal of Clinical Nursing, 2007. **16**(2): p. 407-413.
17. Bossola, M., C. Vulpio, and L. Tazza *Fatigue in chronic dialysis patients*. Seminars in Dialysis, 2011. **24**, 550-555.
18. Kosmadakis, G., et al., *Physical exercise in patients with severe kidney disease.* Nephron Clinical Practice, 2010. **115**(1): p. c7-c16.
19. Picariello, F., et al., *Examining the efficacy of social-psychological interventions for the management of fatigue in End-Stage Kidney Disease (ESKD): A systematic review with meta-analysis.* Health Psychology Review, 2017.
20. Picariello, F., et al., *The role of psychological factors in fatigue among End-Stage Kidney Disease patients: A critical review.* Clinical Kidney Journal, 2017. **10**(1): p. 79-88.
21. Zalai, D., et al., *The importance of fatigue cognitions in chronic hepatitis C infection.* Journal of psychosomatic research, 2015. **78**(2): p. 193-198.
22. Van Kessel, K. and R. Moss-Morris, *Understanding multiple sclerosis fatigue: a synthesis of biological and psychological factors.* Journal of psychosomatic research, 2006. **61**(5): p. 583-585.
23. Alsén, P., et al., *Illness perceptions after myocardial infarction: relations to fatigue, emotional distress, and health-related quality of life.* Journal of Cardiovascular Nursing, 2010. **25**(2): p. E1-E10.
24. Grayson, P.C., et al., *Illness perceptions and fatigue in systemic vasculitis.* Arthritis care & research, 2013. **65**(11): p. 1835-1843.
25. Donovan, K.A., et al., *Utility of a cognitive-behavioral model to predict fatigue following breast cancer treatment.* Health Psychology, 2007. **26**(4): p. 464.
26. Irving, K., et al., *085. Fatigue and Functional Disability in Rheumatoid Arthritis: Evidence for a Cognitive Behavioural Model.* Rheumatology, 2015. **54**(suppl 1): p. i83-i84.
27. Nikolaus, S., et al., *Fatigue and factors related to fatigue in rheumatoid arthritis: a systematic review.* Arthritis care & research, 2013. **65**(7): p. 1128-1146.
28. Gielissen, M.F., et al., *Effects of cognitive behavior therapy in severely fatigued disease-free cancer patients compared with patients waiting for cognitive behavior therapy: A randomized controlled trial.* Journal of Clinical Oncology, 2006. **24**(30): p. 4882-4887.
29. van Kessel, K., et al., *A randomized controlled trial of cognitive behavior therapy for multiple sclerosis fatigue.* Psychosomatic Medicine, 2008. **70**(2): p. 205-213.
30. van den Akker, L.E., et al., *Effectiveness of cognitive behavioral therapy for the treatment of fatigue in patients with multiple sclerosis: A systematic review and meta-analysis.* Journal of Psychosomatic Research, 2016. **90**: p. 33-42.
31. Kangas, M., D.H. Bovbjerg, and G.H. Montgomery, *Cancer-related fatigue: a systematic and meta-analytic review of non-pharmacological therapies for cancer patients.* Psychological bulletin, 2008. **134**(5): p. 700.
32. Beck, A.T., *Cognitive therapy of depression*. 1979: Guilford press.
33. Beck, J.S., *Cognitive behavior therapy: Basics and beyond*. 2011: Guilford Press.
34. Weatherhead, S. and G. Flaherty-Jones, *The Pocket Guide to Therapy: A'how To'of the Core Models*. 2011: Sage.
35. Chalder, T., et al., *Development of a fatigue scale.* Journal of Psychosomatic Research, 1993. **37**(2): p. 147-153.
36. White, P.D., et al., *Comparison of adaptive pacing therapy, cognitive behaviour therapy, graded exercise therapy, and specialist medical care for chronic fatigue syndrome (PACE): a randomised trial.* The Lancet, 2011. **377**(9768): p. 823-836.
37. Dattilio, F.M. and M.A. Hanna, *Collaboration in cognitive-behavioral therapy.* Journal of clinical psychology, 2012. **68**(2): p. 146-158.
38. Horvath, A.O., et al., *Alliance in individual psychotherapy*, 2011, Educational Publishing Foundation.

For peer review only

For peer review only - http://bmjopen.bmj.com/site/about/guidelines.xhtml

39. Heins, M.J., H. Knoop, and G. Bleijenberg, *The role of the therapeutic relationship in cognitive behaviour therapy for chronic fatigue syndrome.* Behaviour research and therapy, 2013. **51**(7): p. 368-376.
40. Hudson, J.L., et al., *Improving distress in dialysis (iDiD): a feasibility two-arm parallel randomised controlled trial of an online cognitive behavioural therapy intervention with and without therapist-led telephone support for psychological distress in patients undergoing haemodialysis.* BMJ open, 2016. **6**(4): p. e011286.
41. Hudson, J.L., et al., *Improving Distress in Dialysis (iDiD): A tailored CBT self-management treatment for patients undergoing dialysis.* Journal of Renal Care, 2016. **42**(4): p. 223-238.
42. Moss-Morris, R., et al., *Protocol for the saMS trial (supportive adjustment for multiple sclerosis): a randomized controlled trial comparing cognitive behavioral therapy to supportive listening for adjustment to multiple sclerosis.* BMC neurology, 2009. **9**(1): p. 45.
43. Everitt, H., et al., *Assessing Cognitive behavioural Therapy in Irritable Bowel (ACTIB): protocol for a randomised controlled trial of clinical-effectiveness and cost-effectiveness of therapist delivered cognitive behavioural therapy and web-based self-management in irritable bowel syndrome in adults.* BMJ open, 2015. **5**(7): p. e008622.
44. Moss-Morris, R., et al., *A pilot randomised controlled trial of an Internet-based cognitive behavioural therapy self-management programme (MS Invigor8) for multiple sclerosis fatigue.* Behaviour Research and Therapy, 2012. **50**(6): p. 415-421.
45. Burgess, M. and T. Chalder, *Overcoming chronic fatigue: A self-help guide using cognitive behavioural techniques.* London: Constable & Robinson Ltd, 2005.
46. Chalder, T., *Coping with chronic fatigue*. 2014: Matrix Audio.
47. Burgess, M. and T. Chalder, *PACE trial: Cognitive Behaviour Therapy for CFS/ME*. 2004: Constable and Robinson.
48. Kazantzis, N., C. Whittington, and F. Dattilio, *Meta-analysis of homework effects in cognitive and behavioral therapy: A replication and extension.* Clinical Psychology: Science and Practice, 2010. **17**(2): p. 144-156.
49. Mausbach, B.T., et al., *The relationship between homework compliance and therapy outcomes: An updated meta-analysis.* Cognitive Therapy and Research, 2010. **34**(5): p. 429-438.
50. Dennison, L. and R. Moss-Morris, *Cognitive–behavioral therapy: what benefits can it offer people with multiple sclerosis?* Expert review of neurotherapeutics, 2010. **10**(9): p. 1383-1390.
51. Roth, A. and S. Pilling, *A competence framework for psychological interventions with people with persistent physical health conditions*, 2015, Retrieved.
52. Eldridge, S.M., et al., *CONSORT 2010 statement: extension to randomised pilot and feasibility trials.* Pilot and feasibility studies, 2016. **2**(1): p. 64.
53. Chilcot, J., et al., *The Chalder Fatigue Questionnaire is a valid and reliable measure of perceived fatigue severity in multiple sclerosis.* Multiple Sclerosis Journal, 2016. **22**(5): p. 677-684.
54. Picariello, F., et al., *Measuring fatigue in haemodialysis patients: The factor structure of the Chalder Fatigue Questionnaire (CFQ).* Journal of Psychosomatic Research, 2016. **84**: p. 81-83.
55. Chalder, T., J. Tong, and V. Deary, *Family cognitive behaviour therapy for chronic fatigue syndrome: An uncontrolled study.* Archives of Disease in Childhood, 2002. **86**(2): p. 95-97.
56. Mundt, J.C., et al., *The Work and Social Adjustment Scale: A simple measure of impairment in functioning.* The British Journal of Psychiatry, 2002. **180**(5): p. 461-464.
57. Chilcot, J., et al., *Psychosocial and Clinical Correlates of Fatigue in Haemodialysis Patients: the Importance of Patients' Illness Cognitions and Behaviours.* International journal of behavioral medicine, 2016. **23**(3): p. 271-281.
58. Kroenke, K. and R.L. Spitzer, *The PHQ-9: a new depression diagnostic and severity measure.* Psychiatric annals, 2002. **32**(9): p. 509-515.

For peer review only

For peer review only - http://bmjopen.bmj.com/site/about/guidelines.xhtml

59. Smarr, K.L. and A.L. Keefer, *Measures of depression and depressive symptoms: Beck Depression Inventory-II (BDI-II), Center for Epidemiologic Studies Depression Scale (CES-D), Geriatric Depression Scale (GDS), Hospital Anxiety and Depression Scale (HADS), and Patient Health Questionnaire-9 (PHQ-9).* Arthritis care & research, 2011. **63**(S11).
60. Kroencke, K., R. Spitzer, and J. Williams, *The PHQ-9: validity of a brief depression severity measure [Electronic version].* Journal of General Internal Medicine, 2001. **16**(9): p. 606-13.
61. Watnick, S., et al., *Validation of 2 depression screening tools in dialysis patients.* American Journal of Kidney Diseases, 2005. **46**(5): p. 919-924.
62. Spitzer, R.L., et al., *A brief measure for assessing generalized anxiety disorder: the GAD-7.* Archives of internal medicine, 2006. **166**(10): p. 1092-1097.
63. Plummer, F., et al., *Screening for anxiety disorders with the GAD-7 and GAD-2: a systematic review and diagnostic metaanalysis.* General hospital psychiatry, 2016. **39**: p. 24-31.
64. Buysse, D.J., et al., *The Pittsburgh Sleep Quality Index: a new instrument for psychiatric practice and research.* Psychiatry research, 1989. **28**(2): p. 193-213.
65. Carpenter, J.S. and M.A. Andrykowski, *Psychometric evaluation of the Pittsburgh sleep quality index.* Journal of psychosomatic research, 1998. **45**(1): p. 5-13.
66. Burkhalter, H., et al., *Structure validity of the Pittsburgh Sleep Quality Index in renal transplant recipients: A confirmatory factor analysis.* Sleep and Biological Rhythms, 2010. **8**(4): p. 274-281.
67. Broadbent, E., et al., *The brief illness perception questionnaire.* Journal of psychosomatic research, 2006. **60**(6): p. 631-637.
68. Weinman, J., et al., *The illness perception questionnaire: a new method for assessing the cognitive representation of illness.* Psychology and health, 1996. **11**(3): p. 431-445.
69. Moss-Morris, R., et al., *The cognitive and behavioral responses to symptoms questionnaire: measuring cognitive and behavioural aspects of symptom interpretation.* preparation. CIT0034, 2009.
70. Skerrett, T.N. and R. Moss-Morris, *Fatigue and social impairment in multiple sclerosis: the role of patients' cognitive and behavioral responses to their symptoms.* Journal of psychosomatic research, 2006. **61**(5): p. 587-593.
71. Mastin, D.F., J. Bryson, and R. Corwyn, *Assessment of sleep hygiene using the Sleep Hygiene Index.* Journal of behavioral medicine, 2006. **29**(3): p. 223-227.
72. Cho, S., G.-S. Kim, and J.-H. Lee, *Psychometric evaluation of the sleep hygiene index: a sample of patients with chronic pain.* Health and quality of life outcomes, 2013. **11**(1): p. 213.
73. Booth, M.L., et al., *International physical activity questionnaire: 12-country reliability and validity.* Med sci sports Exerc, 2003. **195**(9131/03): p. 3508-1381.
74. van Poppel, M.N., et al., *Physical activity questionnaires for adults.* Sports medicine, 2010. **40**(7): p. 565-600.
75. Charlson, M.E., et al., *A new method of classifying prognostic comorbidity in longitudinal studies: development and validation.* J Chronic Dis, 1987. **40**(5): p. 373-83.
76. Di Iorio, B., et al., *Charlson Comorbidity Index is a predictor of outcomes in incident hemodialysis patients and correlates with phase angle and hospitalization.* Int J Artif Organs, 2004. **27**(4): p. 330-6.
77. Jassal, S.V., D.E. Schaubel, and S.S.A. Fenton, *Baseline comorbidity in kidney transplant recipients: a comparison of comorbidity indices.* American journal of kidney diseases, 2005. **46**(1): p. 136-142.
78. Moore, G.F., et al., *Process evaluation of complex interventions: Medical Research Council guidance.* bmj, 2015. **350**: p. h1258.
79. Patton, M.Q., *Two decades of developments in qualitative inquiry a personal, experiential perspective.* Qualitative Social Work, 2002. **1**(3): p. 261-283.

For peer review only - http://bmjopen.bmj.com/site/about/guidelines.xhtml

80. Guest, G., A. Bunce, and L. Johnson, *How many interviews are enough? An experiment with data saturation and variability.* Field Methods, 2006. **18**(1): p. 59-82.
81. Billingham, S.A., A.L. Whitehead, and S.A. Julious, *An audit of sample sizes for pilot and feasibility trials being undertaken in the United Kingdom registered in the United Kingdom Clinical Research Network database.* BMC medical research methodology, 2013. **13**(1): p. 104.
82. Viechtbauer, W., et al., *A simple formula for the calculation of sample size in pilot studies.* Journal of clinical epidemiology, 2015. **68**(11): p. 1375-1379.
83. Browne, R.H., *On the use of a pilot sample for sample size determination.* Statistics in medicine, 1995. **14**(17): p. 1933-1940.
84. Tavakol, M. and R. Dennick, *Making sense of Cronbach's alpha.* International journal of medical education, 2011. **2**: p. 53.
85. Braun, V., V. Clarke, and G. Terry, *Thematic analysis.* Qual Res Clin Health Psychol, 2014: p. 95-114.
86. Senn, B., et al., *Developing and evaluating complex interventions: the new Medical Research Council guidance.* Studies, 2013. **59**: p. 587-592.

For peer review only

For peer review only - http://bmjopen.bmj.com/site/about/guidelines.xhtml

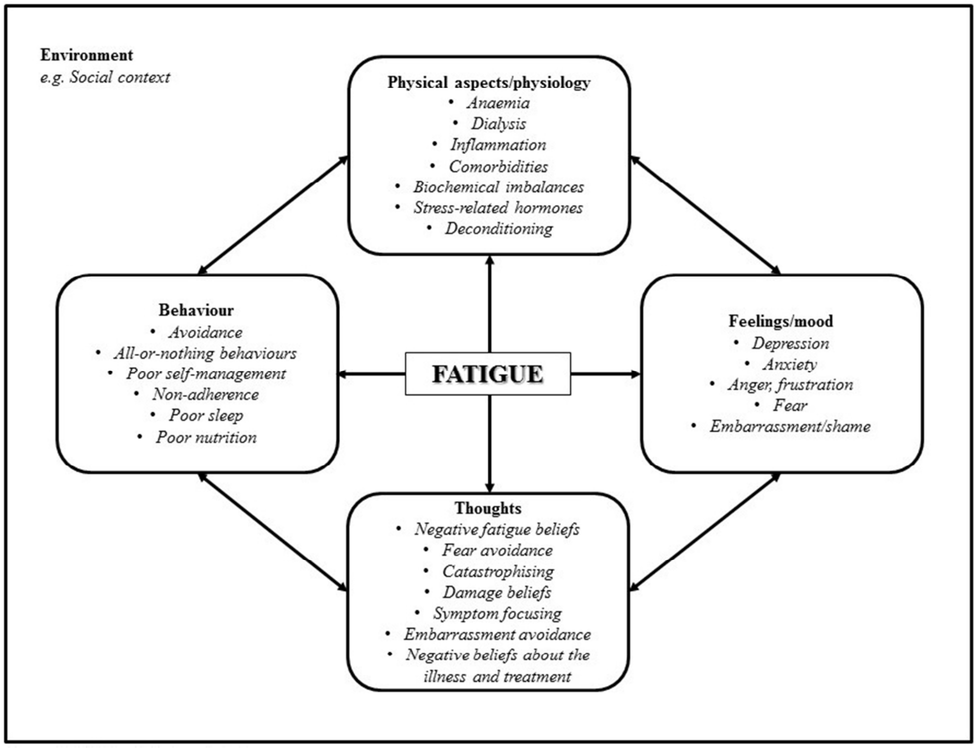

Figure 1. CBT model of renal fatigue.

241x182mm (96 x 96 DPI)

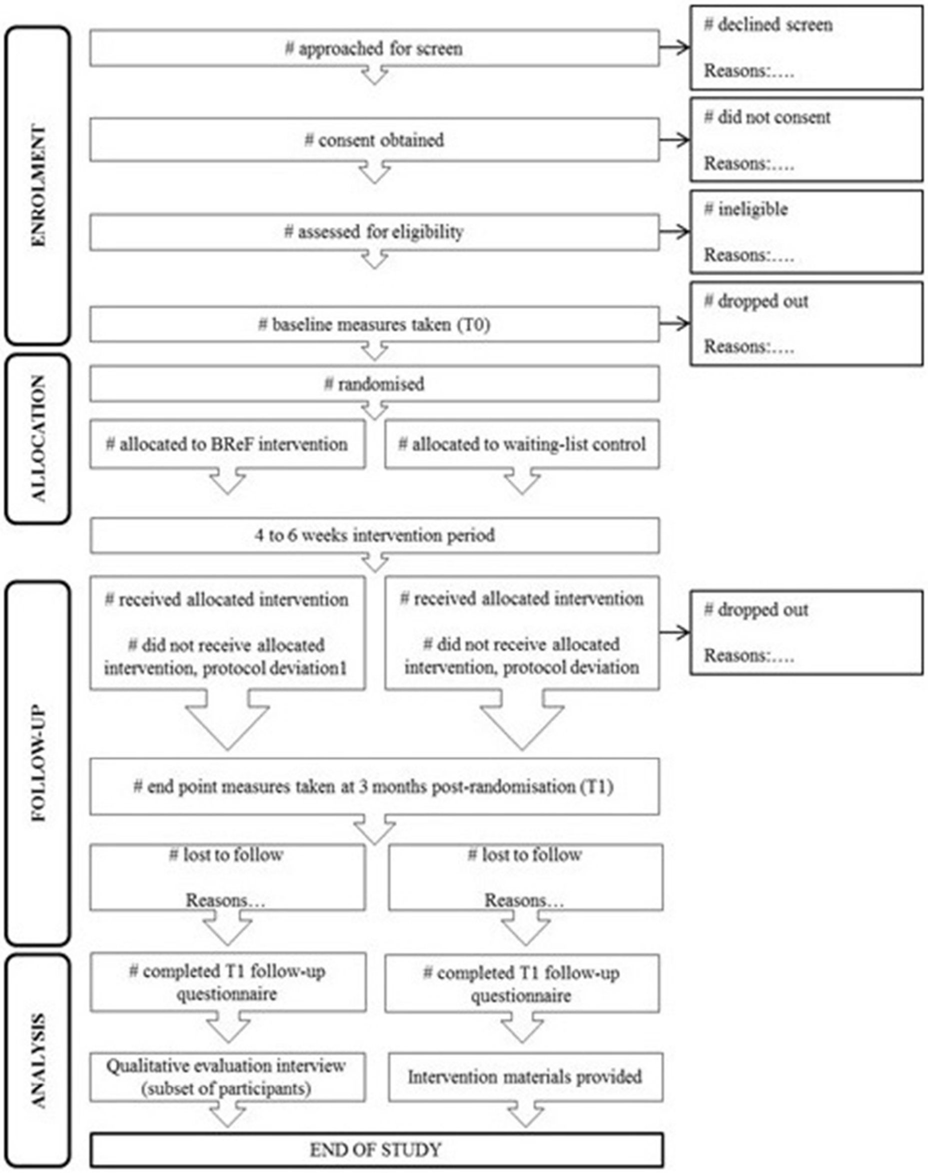

Figure 2. Anticipated flow of participants through the study.

128x163mm (96 x 96 DPI)

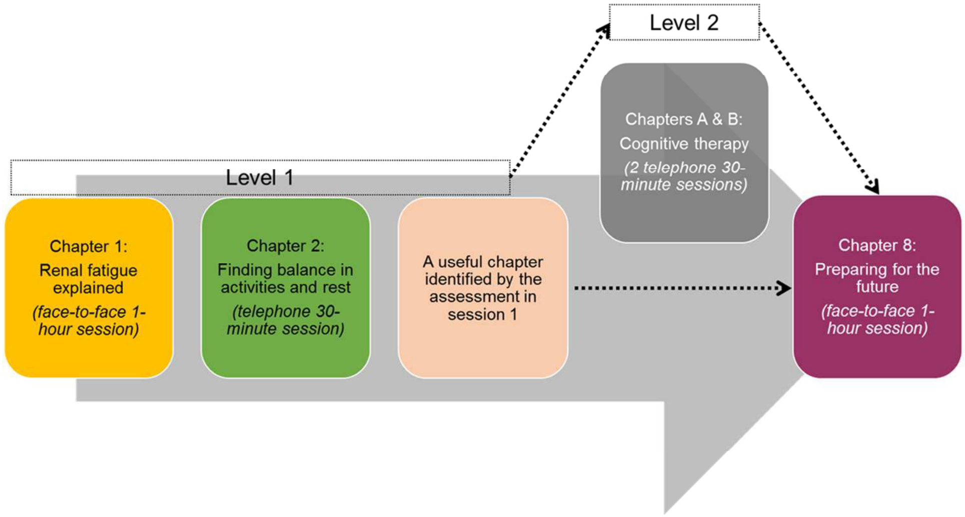

Figure 3. Structure and content of the intervention.

STANDARD PROTOCOL ITEMS: RECOMMENDATIONS FOR INTERVENTIONAL TRIALS

SPIRIT 2013 Checklist: Recommended items to address in a clinical trial protocol and related documents*

Section/item	Item No	Description	Addressed on page number
**Administrative information**			
Title	1	Descriptive title identifying the study design, population, interventions, and, if applicable, trial acronym	page 1
Trial registration	2a	Trial identifier and registry name. If not yet registered, name of intended registry	page 1 (ISRCTN91238019)
	2b	All items from the World Health Organization Trial Registration Data Set	ISRCTN registration form
Protocol version	3	Date and version identifier	page 1
Funding	4	Sources and types of financial, material, and other support	pages 23-24
Roles and responsibilities	5a	Names, affiliations, and roles of protocol contributors	page 1
	5b	Name and contact information for the trial sponsor	ISRCTN registration form
	5c	Role of study sponsor and funders, if any, in study design; collection, management, analysis, and interpretation of data; writing of the report; and the decision to submit the report for publication, including whether they will have ultimate authority over any of these activities	page 23

For peer review only - http://bmjopen.bmj.com/site/about/guidelines.xhtml

	5d	Composition, roles, and responsibilities of the coordinating centre, steering committee, endpoint adjudication committee, data management team, and other individuals or groups overseeing the trial, if applicable (see Item 21a for data monitoring committee)	N/A small feasibility trial
**Introduction**			
Background and rationale	6a	Description of research question and justification for undertaking the trial, including summary of relevant studies (published and unpublished) examining benefits and harms for each intervention	pages 4-5
	6b	Explanation for choice of comparators	page 14
Objectives	7	Specific objectives or hypotheses	pages-5-6
Trial design	8	Description of trial design including type of trial (eg, parallel group, crossover, factorial, single group), allocation ratio, and framework (eg, superiority, equivalence, noninferiority, exploratory)	page 6
**Methods: Participants, interventions, and outcomes**			
Study setting	9	Description of study settings (eg, community clinic, academic hospital) and list of countries where data will be collected. Reference to where list of study sites can be obtained	page 6
Eligibility criteria	10	Inclusion and exclusion criteria for participants. If applicable, eligibility criteria for study centres and individuals who will perform the interventions (eg, surgeons, psychotherapists)	pages 6-7
Interventions	11a	Interventions for each group with sufficient detail to allow replication, including how and when they will be administered	pages 8-13
	11b	Criteria for discontinuing or modifying allocated interventions for a given trial participant (eg, drug dose change in response to harms, participant request, or improving/worsening disease)	N/A
	11c	Strategies to improve adherence to intervention protocols, and any procedures for monitoring adherence (eg, drug tablet return, laboratory tests)	page 15
	11d	Relevant concomitant care and interventions that are permitted or prohibited during the trial	N/A

For peer review only - http://bmjopen.bmj.com/site/about/guidelines.xhtml

Outcomes	12	Primary, secondary, and other outcomes, including the specific measurement variable (eg, systolic blood pressure), analysis metric (eg, change from baseline, final value, time to event), method of aggregation (eg, median, proportion), and time point for each outcome. Explanation of the clinical relevance of chosen efficacy and harm outcomes is strongly recommended	pages 15-19
Participant timeline	13	Time schedule of enrolment, interventions (including any run-ins and washouts), assessments, and visits for participants. A schematic diagram is highly recommended (see Figure)	pages 7-8, Figure 2 and Table 3.
Sample size	14	Estimated number of participants needed to achieve study objectives and how it was determined, including clinical and statistical assumptions supporting any sample size calculations	page 20
Recruitment	15	Strategies for achieving adequate participant enrolment to reach target sample size	pages 9 and 14
**Methods: Assignment of interventions (for controlled trials)**			
Allocation:			
Sequence generation	16a	Method of generating the allocation sequence (eg, computer-generated random numbers), and list of any factors for stratification. To reduce predictability of a random sequence, details of any planned restriction (eg, blocking) should be provided in a separate document that is unavailable to those who enrol participants or assign interventions	page 8
Allocation concealment mechanism	16b	Mechanism of implementing the allocation sequence (eg, central telephone; sequentially numbered, opaque, sealed envelopes), describing any steps to conceal the sequence until interventions are assigned	page 8
Implementation	16c	Who will generate the allocation sequence, who will enrol participants, and who will assign participants to interventions	page 8
Blinding (masking)	17a	Who will be blinded after assignment to interventions (eg, trial participants, care providers, outcome assessors, data analysts), and how	page 8
	17b	If blinded, circumstances under which unblinding is permissible, and procedure for revealing a participant's allocated intervention during the trial	page 8
**Methods: Data collection, management, and analysis**			

Data collection methods	18a	Plans for assessment and collection of outcome, baseline, and other trial data, including any related processes to promote data quality (eg, duplicate measurements, training of assessors) and a description of study instruments (eg, questionnaires, laboratory tests) along with their reliability and validity, if known. Reference to where data collection forms can be found, if not in the protocol	pages 14-17
	18b	Plans to promote participant retention and complete follow-up, including list of any outcome data to be collected for participants who discontinue or deviate from intervention protocols	N/A feasibility trial
Data management	19	Plans for data entry, coding, security, and storage, including any related processes to promote data quality (eg, double data entry; range checks for data values). Reference to where details of data management procedures can be found, if not in the protocol	N/A small feasibility trial
Statistical methods	20a	Statistical methods for analysing primary and secondary outcomes. Reference to where other details of the statistical analysis plan can be found, if not in the protocol	pages 20-22
	20b	Methods for any additional analyses (eg, subgroup and adjusted analyses)	pages 20-22
	20c	Definition of analysis population relating to protocol non-adherence (eg, as randomised analysis), and any statistical methods to handle missing data (eg, multiple imputation)	Feasibility trial, only exploratory analysis
**Methods: Monitoring**			
Data monitoring	21a	Composition of data monitoring committee (DMC); summary of its role and reporting structure; statement of whether it is independent from the sponsor and competing interests; and reference to where further details about its charter can be found, if not in the protocol. Alternatively, an explanation of why a DMC is not needed	No formal DMC, as it is a small feasibility trial, but monthly steering meetings (page 23)
	21b	Description of any interim analyses and stopping guidelines, including who will have access to these interim results and make the final decision to terminate the trial	pages 14 (clinical supervision), 17-18, 23

Harms	22	Plans for collecting, assessing, reporting, and managing solicited and spontaneously reported adverse events and other unintended effects of trial interventions or trial conduct	pages 14 and 18
Auditing	23	Frequency and procedures for auditing trial conduct, if any, and whether the process will be independent from investigators and the sponsor	pages 22-23
**Ethics and dissemination**			
Research ethics approval	24	Plans for seeking research ethics committee/institutional review board (REC/IRB) approval	pages 22-23
Protocol amendments	25	Plans for communicating important protocol modifications (eg, changes to eligibility criteria, outcomes, analyses) to relevant parties (eg, investigators, REC/IRBs, trial participants, trial registries, journals, regulators)	pages 22-23
Consent or assent	26a	Who will obtain informed consent or assent from potential trial participants or authorised surrogates, and how (see Item 32)	Pages 7, 22
	26b	Additional consent provisions for collection and use of participant data and biological specimens in ancillary studies, if applicable	N/A
Confidentiality	27	How personal information about potential and enrolled participants will be collected, shared, and maintained in order to protect confidentiality before, during, and after the trial	pages 22-23
Declaration of interests	28	Financial and other competing interests for principal investigators for the overall trial and each study site	page 24
Access to data	29	Statement of who will have access to the final trial dataset, and disclosure of contractual agreements that limit such access for investigators	page 23 (small feasibility trial)
Ancillary and post-trial care	30	Provisions, if any, for ancillary and post-trial care, and for compensation to those who suffer harm from trial participation	page 23
Dissemination policy	31a	Plans for investigators and sponsor to communicate trial results to participants, healthcare professionals, the public, and other relevant groups (eg, via publication, reporting in results databases, or other data sharing arrangements), including any publication restrictions	page 23
	31b	Authorship eligibility guidelines and any intended use of professional writers	page 23

For peer review only - http://bmjopen.bmj.com/site/about/guidelines.xhtml

	31c	Plans, if any, for granting public access to the full protocol, participant-level dataset, and statistical code	ISRCTN registration: The data sharing plans for the current study are unknown and will be made available at a later date.
**Appendices**			
Informed consent materials	32	Model consent form and other related documentation given to participants and authorised surrogates	Available on request
Biological specimens	33	Plans for collection, laboratory evaluation, and storage of biological specimens for genetic or molecular analysis in the current trial and for future use in ancillary studies, if applicable	N/A

*It is strongly recommended that this checklist be read in conjunction with the SPIRIT 2013 Explanation & Elaboration for important clarification on the items. Amendments to the protocol should be tracked and dated. The SPIRIT checklist is copyrighted by the SPIRIT Group under the Creative Commons "Attribution-NonCommercial-NoDerivs 3.0 Unported" license.

For peer review only

For peer review only - http://bmjopen.bmj.com/site/about/guidelines.xhtml

BMJ Open

Cognitive Behavioural Therapy (CBT) for Renal Fatigue (BReF): A feasibility randomised-controlled trial of CBT for the management of fatigue in haemodialysis (HD) patients.

Journal:	*BMJ Open*
Manuscript ID	bmjopen-2017-020842.R1
Article Type:	Protocol
Date Submitted by the Author:	01-Feb-2018
Complete List of Authors:	Picariello, Federica; King's College London, Health Psychology Section, Psychology Department<br>Moss-Morris, Rona; King's College London, Psychology Department, Insitute of Psychiatry<br>Macdougall, Iain; Kings College Hospital NHS Foundation Trust, Nephrology<br>Norton, Sam; King's College London, Psychology Department, Insitute of Psychiatry<br>Da Silva-Gane, Maria; Lister Hospital, Renal Medicine; University of Hertfordshire<br>Farrington, Ken; Lister Hospital, Renal Medicine; University of Hertfordshire<br>Clayton, Hope; Lister Hospital, Renal Medicine<br>Chilcot, Joseph; King\'s College London, Psychology
**Primary Subject Heading**:	Patient-centred medicine
Secondary Subject Heading:	Mental health, Patient-centred medicine, Qualitative research, Renal medicine
Keywords:	fatigue, Dialysis < NEPHROLOGY, Cognitive behavioural therapy, quality of life, psychotherapy

SCHOLARONE™
Manuscripts

Cognitive Behavioural Therapy (CBT) for Renal Fatigue (BReF): A feasibility randomised-controlled trial of CBT for the management of fatigue in haemodialysis (HD) patients.

v4 29.01.2018

Federica Picariello[1*], Rona Moss-Morris[1], Iain C Macdougall[2], Sam Norton[1], Maria Da Silva-Gane[3,4], Ken Farrington[3,4], Hope Clayton[3], and Joseph Chilcot[1]

[1]Health Psychology Section, Psychology Department, Institute of Psychiatry, Psychology and Neuroscience, King's College London, UK

[2]Department of Renal Medicine, King's College Hospital, London, UK

[3]Department of Renal Medicine, Lister Hospital, Stevenage, UK

[4]University of Hertfordshire, Hertfordshire, UK

* Address for Correspondence

Email: federica.picariello@kcl.ac.uk

Telephone: 02071889324

Postal address: Health Psychology Section, Psychology Department, Institute of Psychiatry, Psychology, and Neuroscience, King's College London, 5th floor Bermondsey Wing, Guy's Campus, London Bridge, London, SE1 9RT, UK

Trial Registration Number: ISRCTN91238019 (pre-results)

Keywords: fatigue; dialysis; cognitive behavioural therapy; kidney failure; quality of life

Word count (excluding tables and reference list): 4797

For peer review only

For peer review only - http://bmjopen.bmj.com/site/about/guidelines.xhtml

Abstract

Introduction: Fatigue is one of the most common and disabling symptoms in End-Stage Kidney Disease (ESKD), particularly among in-centre haemodialysis patients. This two-arm parallel group feasibility randomised-controlled trial will determine whether a fully powered efficacy trial is achievable by examining the feasibility of recruitment, acceptability and potential benefits of a CBT-based intervention for fatigue among in-centre HD patients. **Methods:** We aim to recruit 40 adult patients, undergoing in-centre haemodialysis at secondary care outpatient dialysis units, who meet clinical levels of fatigue. Patients will be randomised individually (using a 1:1 ratio) to either a 4- to 6-week CBT-based intervention (intervention arm) or to a waiting-list control (control arm). The primary feasibility outcomes include descriptive data on numbers within each recruiting centre meeting eligibility criteria, rates of recruitment, numbers retained post-randomisation, and treatment adherence. To assess the potential benefits of the BReF intervention, secondary self-report outcomes include measures of fatigue severity (Chalder Fatigue Questionnaire), fatigue-related functional impairment (Work and Social Adjustment Scale), sleep quality (Pittsburgh Sleep Quality Index), depression (Patient Health Questionnaire-9), and anxiety (Generalised Anxiety Disorder-7). Changes in fatigue perceptions (Brief Illness Perception Questionnaire), cognitive and behavioural responses to fatigue (Cognitive and Behavioural Responses to Symptoms Questionnaire), sleep hygiene behaviours (Sleep Hygiene Index), and physical activity (International Physical Activity Questionnaire –short form) will also be explored. These self-report measures will be collected at baseline and 3 months post-randomisation. Nested qualitative interviews will be conducted post-intervention to explore the acceptability of the intervention and identify any areas in need of improvement. The statistician and assessor will be blinded to treatment allocation. **Ethics and dissemination:** A National Health Service (NHS) research ethics committee approved the study. Any amendments to the protocol will be submitted to the NHS committee and study sponsor. **Trial registration:** ISRCTN91238019 (pre-results).

For peer review only - http://bmjopen.bmj.com/site/about/guidelines.xhtml

Strengths and limitations of this study

- This is the first feasibility trial assessing the feasibility, acceptability, and potential benefits of a psychological intervention for the management of fatigue among in-centre haemodialysis patients.
- The mixed-methods approach will help to evaluate comprehensively and in-depth the feasibility and acceptability of the BReF intervention; the qualitative data will complement the quantitative findings and help to identify areas in need of improvement.
- The BReF intervention was developed systematically, using theory and evidence, with substantial input from patient and public representatives.
- As this is a feasibility trial, it is not powered to detect efficacy.

For peer review only

For peer review only - http://bmjopen.bmj.com/site/about/guidelines.xhtml

Introduction

End-Stage Kidney Disease (ESKD) is a chronic disease of the kidneys, characterised by inadequate renal functioning, where renal replacement therapy (RRT) is necessary to sustain life [1]. Haemodialysis is the most common RRT modality, filtering toxins out of the blood via an artificial extracorporeal blood circuit. A typical HD patient will be required to attend dialysis sessions three times a week for 3-4 hours each time [2]. On average, renal patients experience 14 symptoms, with fatigue emerging as one of the most persistent and debilitating symptoms [3, 4]. Fatigue is a complex and subjective symptom characterized by extreme and persistent tiredness resistant to rest and recuperation [5-7]. Forty-nine to 92% of dialysis patients suffer from fatigue [3]. Fatigue is a substantial contributor to impaired functioning and quality of life, and recent evidence suggests that it has also implications for clinical outcomes [8-14]; yet, it is often under-recognised and under-treated by healthcare professionals, perceived as a normal consequence of the illness and treatment burden [15, 16].

Current management in the form of pharmacological treatments or exercise is ineffective [17, 18] and no theory-driven and evidence-based psychological interventions aimed at fatigue for this group currently exist, although there is some promising evidence for some improvements in fatigue following psychological interventions not aimed at fatigue specifically [19]. As the aetiology of fatigue in renal patients is still largely unknown, no consistent treatment model exists [8, 20].

There is increasing recognition regarding the importance of psychological factors in the perpetuation and maintenance of fatigue in other long-term physical conditions [21-26]. For example, in Multiple Sclerosis (MS), negative fatigue beliefs, such as catastrophizing about the consequences of fatigue, embarrassment about fatigue, and belief that fatigue is a sign of physical damage; and unhelpful behaviours in response to fatigue, like excessive resting or overdoing things followed by long resting periods to recover; were found to be strongly associated with fatigue, above and beyond the role of demographic and clinical factors, such as neurological impairment and remission status [22]. On the other hand, social support may act as a buffer to fatigue perpetuation [27]. An understanding of the contribution of these factors to fatigue has translated into successful psychological interventions, leading to clinically significant improvements in fatigue severity and fatigue-related functional

For peer review only - http://bmjopen.bmj.com/site/about/guidelines.xhtml

impairment [e.g. 28, 29]. There is, in fact, extensive evidence in support of Cognitive Behavioural Therapy (CBT) for adjustment and the management of symptoms, like fatigue, in the context of long-term physical conditions, such as cancer and MS [30, 31], despite being originally developed for the treatment of mood disorders [32, 33]. CBT is a structured, tailored and time-limited talking therapy that focuses on changing negative beliefs and unhelpful behaviours, as well as relaxation techniques, stress-management, and mindfulness to foster resilience [33, 34].

We conducted preliminary work, consisting of prospective and qualitative studies, which revealed the importance of cognitive and behavioural factors in the experience of fatigue in ESKD, in line with the findings from other long-term physical conditions. To date, the effectiveness of CBT specifically for fatigue has not been examined in this population, yet, a similar approach may also be useful here. Based on the findings from these precursor studies and Patient and Public Involvement (PPI) input, we adapted an existing CBT approach initially developed by one of the authors (RMM) for fatigue in Multiple Sclerosis. According to this renal fatigue treatment formulation, which integrates biological and psychosocial factors; there are a number of factors that may act as triggers of fatigue in this patient population, such as anaemia and haemodialysis. Whilst these factors trigger initial symptoms of fatigue, one's thoughts, emotions, and behaviours in response to fatigue may maintain and perpetuate fatigue, potentially leading to a vicious cycle of negative illness and fatigue beliefs, increased distress, and maladaptive behaviours, as displayed in *Figure 1*. The factors maintaining and perpetuating fatigue are targeted in CBT.

Objectives

A feasibility design was deemed necessary to determine whether a full-scale randomised-controlled trial (RCT) is feasible, by considering numbers meeting eligibility criteria, rates of recruitment and retention post-randomisation, floor/ceiling effects that might affect sensitivity to change; as well as by identifying any intervention-specific issues, particularly occurrence of adverse events and timing burden. The following aims will be addressed in this feasibility RCT:

- Objective 1: To estimate rates of recruitment and retention.
- Objective 2: To estimate willingness to be randomised.

For peer review only - http://bmjopen.bmj.com/site/about/guidelines.xhtml

- Objective 3: To explore the level of adherence to the intervention (intervention arm only).
- Objective 4: To estimate the standard deviation of fatigue in this patient population in order to compute a more robust estimate of the sample size required for an efficacy trial.
- Objective 5: To preliminary assess the psychometric properties of the self-report instruments used.
- Objective 6: To explore the potential benefits of the intervention at reducing fatigue severity and fatigue-related functional impairment as compared to the waiting-list control.
- Objective 7: To explore the potential benefits of the intervention at reducing depression and anxiety and improving sleep quality as compared to the waiting-list control.
- Objective 8: To examine change in fatigue-related cognitions and behaviours, and whether their effect differs between the intervention and control arm.
- Objective 9: To qualitatively explore patient perceptions of the acceptability and usefulness of the intervention and identify areas of improvement for a future full-scale trial.
- Objective 10: To explore any intervention-specific issues, particularly setting, mode of delivery of the intervention and acceptable number of sessions/chapters.

Methods

Design

A two-arm parallel group feasibility randomised-controlled trial (RCT). There will be one follow-up assessment at three months post-randomisation. A nested-qualitative study will evaluate patients' experiences with the intervention.

Setting and participants

Outpatient haemodialysis patients will be recruited from two NHS sites in England.

Inclusion and exclusion criteria

Participants are eligible for the study if they:

1. Are over 18 years of age,
2. Have a confirmed ESKD diagnosis

For peer review only - http://bmjopen.bmj.com/site/about/guidelines.xhtml

3. Are experiencing clinical levels of fatigue defined as scoring >18 on the Chalder Fatigue Questionnaire, when using the continuous scoring [35, 36].
4. Have full verbal and written proficiency in English
5. Are receiving in-centre haemodialysis
6. Length of time on dialysis > 90 days
7. Are willing and able to take part in the study and intervention

Patients will be excluded if they:

1. Do not provide informed consent or refuse to be randomised,
2. Have any known cognitive impairments
3. Have a severe mental health disorder, for example, psychosis, bipolar disorder
4. Do not have full verbal and written proficiency in English
5. Are currently receiving psychotherapy
6. Are currently participating in any other intervention trial
7. Are failing on dialysis and approaching end of life (supportive care/palliative care pathway)
8. Have a fatigue (CFQ) score below the cut-off at the pre-randomisation assessment (spontaneous improvement after screening)

Patients will not be screened for anaemia. Levels of haemoglobin and haematocrit are generally maintained within recommended ranges [17]. Additionally, there is evidence for a ceiling effect of anaemia management on fatigue [37] and improvements in fatigue are often below a clinically meaningful threshold, particularly in dialysis patients [38]. Nonetheless, the role of anaemia-related factors will be examined in the exploratory analysis and may lead to changes to the inclusion/exclusion criteria for a future efficacy trial.

Flow of recruitment and participant timeline

Recruitment will take place from October 2017 to July 2018. Patients interested in participating will be given a participant information sheet, screening questionnaire, consisting of sociodemographic and illness-related questions and the Chalder Fatigue Questionnaire; an informed consent form, and a freepost envelope. Potential participants will be given a minimum of 24 hours to establish if they would like to take part. Following consent, eligible participants will receive the baseline questionnaire, and will be randomised after the

For peer review only - http://bmjopen.bmj.com/site/about/guidelines.xhtml

For peer review only

completion of the baseline questionnaire. Participants who score below the fatigue cut-off at the pre-randomization assessment will be excluded.

Participants will be informed of the outcome of the randomisation process over the phone and will receive confirmation of their treatment allocation and materials via post. We anticipate the participant's journey through the study will last approximately 4-5 months, as summarised in *Figure 2*, with approximately one month dedicated to screening and randomisation. The intervention will last between 4 to 6 weeks, depending on each participant's needs. Participants are expected to complete one session per week. Follow-up data will be collected at 3-months post-randomisation (T1). On completion of the post-intervention questionnaire, a subsample of participants will be invited to take part in the qualitative interview. After completion of follow-up assessments at T1, participants in the control condition will receive the intervention manual and tasks workbook via post.

Randomisation, allocation concealment and blinding

Participants will be randomised in a 1:1 ratio to receive either the BReF intervention or to the waiting-list control. Participants will be randomised at the individual level. Randomisation will be stratified by centre and randomly varying block sizes will be used to maintain balance of numbers in each arm across the period of recruitment while maintaining allocation concealment. King's College London's Independent Randomisation Service will be used. Because the randomisation sequence is automated in real time, the allocation sequence is concealed from researchers. The trial coordinator will receive an automated email with the outcome of the randomisation procedure. The nature of the trial is such that blinding of participants cannot be achieved. Follow-up outcomes will be completed independently by participants by post or online. If baseline or follow-up questionnaires are not completed, then participants will receive reminder emails or phone calls and an assistance-based visit at the dialysis unit, by an independent researcher, who has not been involved in the intervention delivery. The statistician will remain blinded to treatment allocation.

BReF Intervention

The intervention is a tailored CBT-based self-management intervention with therapist support. The purpose of this intervention is to target individuals' fatigue beliefs and behaviours in order to facilitate coping with ReF. Further detail on the intervention can be

For peer review only - http://bmjopen.bmj.com/site/about/guidelines.xhtml

found in *Figure 3*. Therapist guidance was deemed necessary to facilitate engagement with the programme, particularly in the formulation of a personal biopsychosocial model of fatigue and identification of unhelpful thoughts and behaviours [39-41].

The development of the CBT-based intervention was systematic, based on the findings of reviews and qualitative and prospective studies, with substantial input from 10 patient and public representatives and a multidisciplinary team of health psychologists, clinical psychologists, and nephrologists. The structure and content of the manual was drafted based on previous CBT interventions developed by one of our authors (RMM), such as "*Managing Your Multiple Sclerosis Fatigue: A Cognitive behavioural therapy manual*" and "*Improving Distress in Dialysis*"[42-46] and other sources [47-49].

Participants will be provided with a structured CBT manual and a tasks workbook, including goal-setting sheets. This will be accompanied by three to five sessions with either a primary researcher who has a background in Health Psychology, basic CBT training and experience in working with fatigued patient groups (FP), or a Registered Health Psychologist working in the renal setting (HC). In accordance with CBT principles, participants will be encouraged to complete tasks between sessions. Completion of these tasks has been found to be predictive of CBT outcomes [50, 51].

The manual consists of 10 chapters, accompanied by a tasks workbook for each session. Please see Table 1 for the content of each chapter and associated tasks to be completed between sessions. The programme consists of two units, the basic unit (Level 1) or the advanced unit (Level 2). In the basic unit, participants will cover 4 chapters out of the manual, three will be accompanied by therapist sessions and one will be selected according to participants' needs, established in the assessment. Participants, engaging well in the first two sessions, will be given the opportunity to cover an additional 2 chapters with the therapist over the phone (Level 2). Engagement will be discussed in clinical supervision, and will be assessed through, for example, completion of between session tasks and focus maintained during the sessions. The brief and stepped structure of the intervention was chosen to meet patients' needs, particularly their fatigability and potential concentration difficulties, according to the practical considerations previously raised with regards to the delivery of CBT in MS [52]. Sessions will be guided by participants' needs, identified through the self-monitoring tasks.

For peer review only - http://bmjopen.bmj.com/site/about/guidelines.xhtml

Table 1

Summary of the Content of the BReF Manual

Chapter	Content	Between sessions task
**Renal fatigue explained**	Understanding ReF and alternative explanations<br>A model for renal fatigue<br>Assessment of fatigue	Fatigue self-monitoring
**Finding balance in activities and rest**	Patterns of rest and activity and its effects on the body<br>Planning activity and rest<br>Exercise	Activity difficulty task<br>Activity and rest goal sheet
**Improving sleep**	Sleep hygiene<br>Maladaptive sleep patterns<br>Improving sleep	Sleep, activity, and rest goal sheet
**Learning to relax**	Diaphragmatic breathing<br>Progressive Muscle Relaxation (PMR)<br>Relaxation training: step-by-step	Relaxation diary
**Coping with emotions**	Strategies to cope with negative emotions<br>Self-assessment of negative emotions<br>Expressing emotions	Coping with negative emotions goal sheet
**Managing stress**	General tips to reduce the impact stress has on your life<br>Managing controllable and uncontrollable stressors<br>Mindfulness	Managing stress goal sheet

For peer review only - http://bmjopen.bmj.com/site/about/guidelines.xhtml

**Making use of your social support**	Creating a support network<br>Disclosure vs. keeping it to self<br>Social comparisons	Social support goal sheet
**Becoming aware of your thinking**	Common unhelpful thoughts<br>Identifying unhelpful thinking	Thought record
**Changing your thinking**	Identifying alternative thoughts	Alternative thoughts goal sheet
**Preparing for the future**	Sustaining and building upon improvements<br>Developing future goals<br>Tips for everyday life	Long-term goals worksheet

For peer review only - http://bmjopen.bmj.com/site/about/guidelines.xhtml

The first and last sessions will be face-to-face lasting one hour, while the remaining sessions will be over the phone, and will last 30 minutes. A combination of face-to-face and telephone sessions has been previously suggested for CBT in MS, to overcome possible limitations of individual delivery methods [52]. Sessions will be scheduled at times that suit the participants. Face-to-face sessions will be conducted in a private environment. For the telephone sessions, participants will be encouraged to have the sessions in a quiet and private environment and allocate sufficient time not to feel rushed. Please see the TIDieR checklist for a summary (Table 2).

For peer review only

For peer review only - http://bmjopen.bmj.com/site/about/guidelines.xhtml

Table 2

TIDieR Checklist Summary

Item 1: Brief name	Item 2: Rationale	Item 3: Materials	Item 4: Procedure	Item 5: Who provided	Item 6: Delivery mode	Item 7: Location/Setting	Item 8: When and how much	Item 9: Tailoring	Item 10: How well (planned)
CBT for Renal Fatigue (BReF)	Please see pages 11-15	BReF manual and tasks workbook	Please see pages 21-24	Primary researcher who has a background in Health Psychology, and experience in working with fatigued patient groups OR Registered Health Psychologist working in the renal setting.	All sessions individual:<br>-2 sessions face-to-face<br>-1 to 3 sessions over the phone	Recruitment from outpatient haemodialysis units in the UK.<br><br>Therapy sessions in a private environment	3 to 5 weekly sessions with the therapist, depending on engagement.<br><br>2 sessions face-to-face, lasting 60 minutes<br><br>1 to 3 sessions over the phone, lasting 30 minutes<br>1 session completed independently	Yes, tailored: optional session determined through the assessment in session 1. Tailored to participants' needs, identified through the self-monitoring.	Therapists will follow a structured intervention manual. Therapy sessions will be audio-recorded and assessed for fidelity by the supervisor, RMM.

For peer review only - http://bmjopen.bmj.com/site/about/guidelines.xhtml

Waiting-list control (control arm)

Participants allocated to the control arm of the study will receive their usual renal care, consisting of attending dialysis. As part of this feasibility trial, what constitutes usual care will be monitored to determine a control arm for a future efficacy trial and to handle potential contamination between the arms. After completion of the follow-up questionnaire, participants in the control group will receive the intervention manual and tasks workbook, but will not receive therapist support sessions. To minimise attrition from the control group, participants will be called to remind them that they can gain access to the program in the weeks that follow.

Clinical supervision

FP and HC will receive training on how to deliver the therapist support sessions from RMM, with approximately 3 to 4 hours of face-to-face contact, in addition to audio-recorded role-playing sessions with feedback. FP and HC will receive continuous supervision throughout the intervention from RMM, following the framework developed to support the delivery of psychological therapies with persistent physical conditions [53]. This will involve reflection and discussion of the sessions, feedback on the audio-recorded sessions and case management, particularly following the initial session with each participant in terms of the treatment plan and subsequently discussions around each participant's progress over the course of the intervention and progression to the Level 2 sessions.

Intervention fidelity

Two therapists will deliver all the intervention sessions following the detailed and structured manual developed for the patients. With permission from the participants, sought on the consent form, therapy sessions will be audio-recorded and a random sample assessed for fidelity during supervision by RMM.

Data collection and feasibility outcomes

The primary focus of this trial is the feasibility of the BReF intervention.

Primary feasibility outcomes

Feasibility will be assessed by collecting descriptive data on recruitment and retention rates and willingness to be randomised according to CONSORT feasibility and pilot trial

For peer review only - http://bmjopen.bmj.com/site/about/guidelines.xhtml

guidelines [54]. In the intervention arm, the degree of adherence to the intervention will also be assessed by recording completion of the chapter selected in session 1 and completed by participants independently in week 3, and the between sessions tasks, as well as recording of attendance at therapist sessions (i.e. did not attend (DNA's) and adherence to the assigned session time). Uptake of and adherence to the Level 2 sessions will also be recorded. Given the exploratory nature of this trial, the number of completed intervention components will be assessed, this may help to identify an adherence cut-off for a future efficacy trial.

Secondary self-reported patient outcomes

Self-reported patient outcomes will also be collected at baseline (T0) and 3-months post-randomisation (T1) via post or online. The assessment schedule completed by patients is summarised in Table 3 and the self-report instruments used are described below:

<u>Fatigue severity</u>

Chalder Fatigue Questionnaire (CFQ)[35]. This instrument measures fatigue severity via 11-items scored against a four-point Likert-type response scale. Scores are assigned for each response, using continuous scoring from 0 to 3. A cut-off of greater than 18 defines a fatigue case [35, 36]. Higher scores represent greater fatigue severity. The total score will be used here following recent psychometric evidence [55, 56]. This scale displays excellent psychometric properties [35, 57] and has been validated among HD patients [56].

<u>Fatigue-related functional impairment</u>

Work and Social Adjustment Scale (WSAS)[58]. The scale consists of five items that correspond to impairment in work, home management, social activities, private leisure activities and relationships as consequence of an illness or symptom, in this case fatigue. Higher scores indicate greater impairment. It has good psychometric properties [58] and has been previously used with HD patients [59].

<u>Depression</u>

Patient Health Questionnaire-9 (PHQ-9)[60]. This instrument measures depression over the last two weeks via a 9-items scale and an additional item to assess the impact of depression on functioning. Each item is scored from 0 (not at all) to 3 (nearly every day), with greater scores representing greater severity of depression. Depression severity cut-offs are available.

For peer review only - http://bmjopen.bmj.com/site/about/guidelines.xhtml

The functional item is rated from "Not at all difficult" to "Extremely difficult". The PHQ-9 displays excellent psychometric properties and is responsive to change [61, 62]. The PHQ-9 has been validated in haemodialysis [63].

Anxiety

Generalised Anxiety Disorder-7 (GAD-7)[64]. This instrument measures anxiety over two weeks, via a 7-items scale, and an additional item to assess the impact of anxiety on functioning. Each item is scored from 0 (not at all) to 3 (nearly every day), with greater scores representing greater severity of anxiety. Anxiety severity cut-offs are available. The functional item is rated from "Not at all difficult" to "Extremely difficult". The PHQ-9 displays excellent psychometric properties [64, 65]. This instrument has been used across chronic conditions, including haemodialysis patients [e.g. 42].

Sleep quality

Pittsburgh Sleep Quality Index (PSQI)[66]. This instrument measures 7 components of sleep quality (subjective sleep quality, sleep latency, sleep duration, sleep efficiency, sleep disturbance, use of sleep medication, daytime dysfunction) over a one-month time interval, via 19-items. Items are scored on an interval scale from 0 to 3. The scores of the components are then summed to obtain a global sleep quality score, ranging from 0 to 21. Higher scores indicate worse sleep quality. This scale displays satisfactory psychometric quality across patient populations [66, 67] and is widely used and has been validated [68].

Process variables

Fatigue perceptions

Brief Illness Perceptions Questionnaire (BIPQ) [69]. This instrument relies on a single-item approach to measure fatigue perceptions. It is a shorter version of the original Illness Perception Questionnaire (IPQ)[70], with moderate to good associations between the two [69]. Five of the items assess cognitive illness/symptom representations (consequences, timeline, personal control, treatment control and identity), two of them assess emotional representation (concern and emotions) and one item assesses illness/symptom comprehension. The items are rated using a response scale of 0 to 10. The psychometric properties of this measure have been assessed using samples from several illness groups, including renal disease [69], displaying satisfactory quality.

For peer review only - http://bmjopen.bmj.com/site/about/guidelines.xhtml

Cognitive and behavioural responses to fatigue

Cognitive and Behavioural Responses to Symptoms Questionnaire (CBSQ) [71]. The scale includes five cognitive subscales; fear avoidance, embarrassment avoidance, catastrophizing about symptoms, beliefs that symptoms signal damage to the body (damage beliefs), and symptom focus. There are also two behavioural subscales; resting and avoidance of activity and all-or-nothing behaviour. All items are scored on a five-point frequency scale ranging from never (0) to all the time (4). Item scores are added from each subscale to obtain a total score. Across studies, this instrument displays acceptable psychometric quality [72] and it has been used with different patient populations, including HD patients [59].

Sleep hygiene behaviours

Sleep Hygiene Index (SHI)[73]. This instrument measures sleep-related behaviours via 13 items. Each item is rated on a five-point scale, ranging from 0 (never) to 4 (always). Total scores range from 0 to 52, with higher scores representing poorer sleep hygiene. This scale displays adequate reliability and validity [73, 74]. However, it has not yet been validated in kidney failure.

Physical activity

International Physical Activity Questionnaire –short form (IPAQ-SF)[75]. This instrument measures self-reported weekly time spent on physical activities (walks, physical exertion of moderate and vigorous intensities) and inactivity (sitting) via 7 items. The questionnaire can be scored categorically according to developed cut-offs to classify individuals into low, moderate, or high physical activity groups; or it can be scored continuously. Responses can be converted to Metabolic Equivalent Task minutes per week (METmin/wk), according to the IPAQ scoring protocol. MET scores across the three sub-components can be summed to indicate overall physical activity [75]. The IPAQ is the most widely validated questionnaire, however, with some inconsistent evidence on its reliability and validity [76]. Given its brevity, simplicity, and extensive use across research, it was selected here to measure physical activity.

Adverse events

Information about occurrence of serious adverse events since the start of the study will be collected by self-report post-intervention, according to good clinical practice

For peer review only - http://bmjopen.bmj.com/site/about/guidelines.xhtml

guidelines. Adverse events will be flagged up to the trial management team and participants will be contacted to further assess the adverse event and its relationship to the study.

Other treatments

Participants will be asked whether they have received any pharmacological, psychological, or exercise-based treatment for depression and/or anxiety and/or fatigue in addition to BReF since starting the study.

Table 3

Schedule of Assessments

	TIME		
**Assessment**	Screening	Baseline (T0)	Post-intervention (T1)
**CFQ**	x	x	x
**WSAS**		x	x
**PHQ-9**		x	x
**GAD-7**		x	x
**PSQI**		x	x
**BIPQ**		x	x
**CBSQ**		x	x
**SHI**		x	x
**IPAQ-short**		x	x
**CCI**		x	
**Sociodemographic characteristics**	x	x	
**Clinical characteristics**	x	x	
**Biochemical outcomes**		x	x
**Self-reported adverse events**			x
**Self-reported treatments for distress or fatigue during study**			x
**Qualitative interviews**			x

For peer review only - http://bmjopen.bmj.com/site/about/guidelines.xhtml

Demographic, social-situational and clinical characteristics

At baseline, socio-demographic characteristics, including: gender, age, ethnicity, marital status, employment status, education, living arrangements, exercise, smoking status, and alcohol consumption; and clinical characteristics, including: dialysis vintage and receipt of anaemia treatments will be collected via self-report.

Extra renal comorbidity will be assessed at baseline by consultant nephrologists using the Charlson Comorbidity Index (CCI) [77]. This instrument is a weighted index that takes into account the number and the seriousness of comorbid diseases and is adjusted for age. The method of classifying comorbidity provides a simple, readily applicable and valid method of estimating risk of death from comorbid disease for use in longitudinal studies [77]. The CCI has been previously used with and determined suitable for dialysis patients [78, 79].

Clinical information, including: dialysis adequacy (Urea Reduction Ratio), inter-dialytic weight gain, haemoglobin, ferritin, serum albumin, creatinine, urea, phosphate, potassium, calcium, CRP, and primary renal diagnosis will be extracted from patients' medical notes at baseline (T0) and post-intervention (T1).

Qualitative interviews

To complement the quantitative process evaluation and further explore the acceptability of the intervention, in line with current MRC process evaluation guidelines [80], qualitative interviews will be conducted with a subgroup of participants from the intervention group at 3-months post-randomization (T1). The interviews will be semi-structured and will be conducted over the phone or face-to-face, in a private environment. The interviews will be conducted by an independent researcher, who has not been involved in the intervention delivery. The main aim of the interviews will be to gather participants' experiences of the intervention, to identify areas of improvement. Purposive maximum variation sampling will be employed to ensure variability of the sample across a range of sociodemographic and clinical characteristics [81], in particular: age, gender, ethnicity, dialysis vintage, degree of adherence to the intervention, degree of improvements in outcomes following the intervention. A minimum of 10 interviews will be conducted, until data saturation is reached, meaning the point where no new data is obtained with every new interview [82].

For peer review only - http://bmjopen.bmj.com/site/about/guidelines.xhtml

Sample size

The renal service of KCH has approximately 550 HD patients and Lister Hospital has approximately 510 HD patients, in which we expect to be able to approach 636 (60%) during the recruitment period. Past psychological research in dialysis patients, conducted by the team, suggest consent rates between 50-70%, assuming a more conservative uptake of 40%, 254 patients are expected to be screened, with approximately 30% (N=76) expected to meet the inclusion criteria, including around half reporting clinical levels of fatigue [59]. Assuming 50% of those eligible will consent to be randomised (N=38), a sample size of 40 participants would allow us to estimate the true population consent rate with a 11% margin of error (95% binomial exact confidence level) for those meeting eligibility criteria. In line with recommended sample sizes of pilot feasibility trials [83-85], 40 patients is deemed sufficient to explore feasibility, acceptability, and potentially efficacy of the intervention, assuming retention rates of 80%, the true population consent rate will be with a margin of error of 13% (95% binomial exact confidence interval) – an acceptable level of error, based on the time, budget and workforce constraints, as FP will act as both the trial coordinator and therapist.

Analysis plan

Descriptive statistics of patients approached, screened, eligible, consented, and randomised will be computed to address Objectives 1-2. Reasons for non-consent, exclusion, and drop-out, at each stage of the study, will be recorded and reported. Adherence to the intervention will be reported using descriptive statistics to address Objective 3. The following values will be computed: mean number of homework tasks completed, mean number of sessions completed, a breakdown on the number of participants completing each session, number of participants completing the independent chapter, mean duration of the telephone and face-to-face sessions. Standard deviations by trial arm will be computed for the fatigue outcomes in order to estimate a more robust sample size for a future efficacy trial, thereby, addressing Objective 4.

The psychometric quality of the self-report instruments used will be assessed to address Objective 5. Reliability will be assessed using Cronbach's alpha, with a minimum acceptable cut-off at $\alpha = 0.70$, but preferably at $\alpha = 0.80$ or higher, particularly for the key variables [86] and individual items will be checked to ensure that there are no problematic items for this patient population. Convergent validity will be assessed via Pearson's correlations between psychological constructs (e.g. depression and fatigue severity) and

For peer review only - http://bmjopen.bmj.com/site/about/guidelines.xhtml

clinical markers (e.g. comorbidities and fatigue severity). Additionally, content validity of the fatigue measures will be considered based on the qualitative data to ensure that the selected measures capture changes described by participants. Responsiveness/sensitivity to change will be assessed by checking correlations between change scores on key variables of interest and by triangulation with patients' narratives.

Given the feasibility nature of the trial, statistical significance will not be assessed; instead effect sizes and confidence intervals will be estimated. An ANCOVA will be performed to estimate the post-intervention mean difference in outcomes, controlling for the baseline levels of each outcome, for the following variables: fatigue severity, fatigue-related functional impairment, depression, anxiety and subjective sleep quality, thereby addressing Objectives 6 and 7. Group allocation will be included as an indicator variable following the intention-to-treat principle. Recruitment centre will also be controlled for in the analysis as it is a stratification factor. Differences in intervention effects by sociodemographic and clinical factors on fatigue outcomes will be explored.

Changes in fatigue perceptions, and cognitions and behaviours in response to fatigue will be evaluated via ANCOVAs to address Objective 8. The proportion of the treatment effect that may be accounted for by these process variables with confidence intervals will also be calculated, as there will be insufficient power for mediation analyses.

To meet Objective 9 and qualitatively explore the acceptability and usefulness of the intervention from the perspective of the participants, the semi-structured qualitative interviews will be transcribed verbatim and analysed using inductive thematic analysis with the use of *NVivo* software. Thematic analysis revolves around identifying recurrent themes and patterns from the interviews and developing a coding manual [87].

To address Objective 10, a mixed methods approach will be used, drawing on both the quantitative and qualitative findings to determine any intervention-specific issues, such as the optimal number of sessions.

Considering issues relating to recruitment and retention rates, suitability of the selected measures, as well as any intervention-specific issues, will help us to determine whether to proceed to a full-scale efficacy trial, if so, these findings will also inform aspects

For peer review only - http://bmjopen.bmj.com/site/about/guidelines.xhtml

of the design of the efficacy trial, such as the required sample size and appropriate self-report measures to ensure sufficient power and sensitivity to detect any intervention effects.

Discussion

Fatigue is common in chronic haemodialysis (HD) patients with consequences on patients' functioning and daily living, as well as implications on clinical outcomes. BReF is a theory-driven and evidence-based CBT intervention with therapist support aimed at improving renal fatigue, that has been designed following the MRC guidance for developing and evaluating complex interventions [88].

This is the first feasibility RCT to examine whether a fatigue-specific CBT-based programme with therapist support is feasible, acceptable, and possibly beneficial at reducing fatigue severity and fatigue-related functional impairment in fatigued patients undergoing in-centre HD. The brief and stepped structure of the intervention may be more appropriate for patients receiving in-centre HD. Prior to proceeding to a full-scale trial, it is important to identify unique challenges with recruitment and retention in this particular setting and to explore whether the content and structure of the manual are deemed useful and relevant by patients. The results of the BReF trial will inform the design of a future full-scale trial powered to detect the efficacy of CBT for the management of fatigue in HD, accompanied by a longer follow-up to assess any sustained effects of the intervention on outcomes.

Ethics

The trial received ethical approval from the London Bridge NHS Research Ethics Committee (17/LO/1406) and is co-sponsored by King's College London and King's College Hospital NHS Foundation Trust. Patient consent will be obtained. The Chief Investigator (CI) and all members of the research team will preserve the confidentiality of participants taking part in the study and will work in accordance with the Caldicott Principles, Data Protection Act 1998, NHS Code of Confidentiality and any relevant NHS Trust organisational policies. All serious adverse events related to the study will be reported to the study sponsor, ethics committee and relevant NHS R&D departments. Authorisation will be sought from the study sponsor for any future substantial and non-substantial amendments arising during the course of the study, prior to submission to the HRA. The study may be subject to inspection and audit by King's College London under their remit as sponsor and other regulatory bodies to

For peer review only - http://bmjopen.bmj.com/site/about/guidelines.xhtml

ensure adherence to GCP and the NHS Research Governance Framework for Health and Social Care (2nd edition).

Indemnity

The study is co-sponsored by King's College London and King's College Hospital, providing insurance for the study, through its own professional indemnity for research involving human participants and no fault compensation and the Trust having a duty of care to patients via NHS indemnity cover, in respect of any claims arising as a result of clinical negligence by its employees, brought by or on behalf of a study patient.

Dissemination

We will endeavour to publish the findings of this trial in a peer-reviewed journal and present the findings at relevant national and international conferences.

Contributors

All authors (FP, RMM, ICM, SN, MDS-G, KF, HC, JC) contributed to the design of the study and writing of the protocol. FP, RMM, JC, ICM were involved in the development of the BReF intervention. FP, JC, and SN were involved in the statistical analysis plan.

The day-to-day management of the study will be co-ordinated by the CI and the trial management team. The team will also meet regularly, once a month, to discuss the overall running of the study, including: rates of recruitment, adherence to the protocol, and safety of patients.

Trial Status

The study will start recruitment at the end of October 2017. Recruitment will continue until July 2018. Patient involvement in the study will conclude in November 2018.

Funding

This work is embedded within a larger PhD project funded by a Biomedical Research Studentship to Miss Federica Picariello from the National Institute for Health Research (NIHR) Biomedical Research Centre at South London and Maudsley NHS Foundation Trust and King's College London. The views expressed are those of the authorsand not necessarily those of the NHS, the NIHR or the Department of Health.

For peer review only - http://bmjopen.bmj.com/site/about/guidelines.xhtml

For peer review only

Conflict of interest

We hereby declare that to our knowledge there are no conflicts of interest. This work has not been published previously in whole or part.

References

1. Haynes, R.J. and C.G. Winearls, *Chronic kidney disease.* Surgery (Oxford), 2010. **28**(11): p. 525-529.
2. Mactier, R., N. Hoenich, and C. Breen, *Haemodialysis.* . 2009, The Renal Association.
3. Almutary, H., A. Bonner, and C. Douglas, *Symptom burden in chronic kidney disease: A review of recent literature.* Journal of Renal Care, 2013. **39**(3): p. 140-150.
4. Murtagh, F.E., et al., *Symptoms in advanced renal disease: a cross-sectional survey of symptom prevalence in stage 5 chronic kidney disease managed without dialysis.* Journal of Palliative Medicine, 2007. **10**(6): p. 1266-1276.
5. Cella, D., et al., *Progress toward guidelines for the management of fatigue.* Oncology (Williston Park, NY), 1998. **12**(11A): p. 369-377.
6. Dittner, A.J., S.C. Wessely, and R.G. Brown, *The assessment of fatigue: a practical guide for clinicians and researchers.* Journal of psychosomatic research, 2004. **56**(2): p. 157-170.
7. Ream, E. and A. Richardson, *Fatigue: a concept analysis.* International journal of nursing studies, 1996. **33**(5): p. 519-529.
8. Artom, M., et al., *Fatigue in advanced kidney disease.* Kidney International, 2014. **86**(3): p. 497-505.

For peer review only

For peer review only - http://bmjopen.bmj.com/site/about/guidelines.xhtml

9. Jhamb, M., et al., *Correlates and outcomes of fatigue among incident dialysis patients.* Clinical Journal of the American Society of Nephrology, 2009. **4**(11): p. 1779-1786.

10. Jhamb, M., et al., *Impact of fatigue on outcomes in the hemodialysis (HEMO) study.* American Journal of Nephrology, 2011. **33**(6): p. 515-523.

11. Koyama, H., et al., *Fatigue is a predictor for cardiovascular outcomes in patients undergoing hemodialysis.* Clinical Journal of the American Society of Nephrology, 2010. **5**(4): p. 659-666.

12. Davison, S.N. and G.S. Jhangri, *Impact of pain and symptom burden on the health-related quality of life of hemodialysis patients.* Journal of Pain and Symptom Management, 2010. **39**(3): p. 477-485.

13. Bonner, A., M. Caltabiano, and L. Berlund, *Quality of life, fatigue, and activity in Australians with chronic kidney disease: A longitudinal study.* Nursing & Health Sciences, 2013. **15**(3): p. 360-367.

14. Bossola, M., et al., *Fatigue Is Associated with Increased Risk of Mortality in Patients on Chronic Hemodialysis.* Nephron, 2015. **130**(2): p. 113-118.

15. Weisbord, S.D., et al., *Renal provider recognition of symptoms in patients on maintenance hemodialysis.* Clinical Journal of the American Society of Nephrology, 2007. **2**(5): p. 960-967.

16. Lee, B.O., et al., *The fatigue experience of haemodialysis patients in Taiwan.* Journal of Clinical Nursing, 2007. **16**(2): p. 407-413.

17. Bossola, M., C. Vulpio, and L. Tazza *Fatigue in chronic dialysis patients*. Seminars in Dialysis, 2011. **24**, 550-555.

18. Kosmadakis, G., et al., *Physical exercise in patients with severe kidney disease.* Nephron Clinical Practice, 2010. **115**(1): p. c7-c16.

For peer review only

19. Picariello, F., et al., *Examining the efficacy of social-psychological interventions for the management of fatigue in End-Stage Kidney Disease (ESKD): A systematic review with meta-analysis.* Health Psychology Review, 2017.

20. Picariello, F., et al., *The role of psychological factors in fatigue among End-Stage Kidney Disease patients: A critical review.* Clinical Kidney Journal, 2017. **10**(1): p. 79-88.

21. Zalai, D., et al., *The importance of fatigue cognitions in chronic hepatitis C infection.* Journal of psychosomatic research, 2015. **78**(2): p. 193-198.

22. Van Kessel, K. and R. Moss-Morris, *Understanding multiple sclerosis fatigue: a synthesis of biological and psychological factors.* Journal of psychosomatic research, 2006. **61**(5): p. 583-585.

23. Alsén, P., et al., *Illness perceptions after myocardial infarction: relations to fatigue, emotional distress, and health-related quality of life.* Journal of Cardiovascular Nursing, 2010. **25**(2): p. E1-E10.

24. Grayson, P.C., et al., *Illness perceptions and fatigue in systemic vasculitis.* Arthritis care & research, 2013. **65**(11): p. 1835-1843.

25. Donovan, K.A., et al., *Utility of a cognitive-behavioral model to predict fatigue following breast cancer treatment.* Health Psychology, 2007. **26**(4): p. 464.

26. Irving, K., et al., *085. Fatigue and Functional Disability in Rheumatoid Arthritis: Evidence for a Cognitive Behavioural Model.* Rheumatology, 2015. **54**(suppl 1): p. i83-i84.

27. Nikolaus, S., et al., *Fatigue and factors related to fatigue in rheumatoid arthritis: a systematic review.* Arthritis care & research, 2013. **65**(7): p. 1128-1146.

28. Gielissen, M.F., et al., *Effects of cognitive behavior therapy in severely fatigued disease-free cancer patients compared with patients waiting for cognitive behavior*

For peer review only

For peer review only - http://bmjopen.bmj.com/site/about/guidelines.xhtml

therapy: A randomized controlled trial. Journal of Clinical Oncology, 2006. **24**(30): p. 4882-4887.

29. van Kessel, K., et al., *A randomized controlled trial of cognitive behavior therapy for multiple sclerosis fatigue.* Psychosomatic Medicine, 2008. **70**(2): p. 205-213.

30. van den Akker, L.E., et al., *Effectiveness of cognitive behavioral therapy for the treatment of fatigue in patients with multiple sclerosis: A systematic review and meta-analysis.* Journal of Psychosomatic Research, 2016. **90**: p. 33-42.

31. Kangas, M., D.H. Bovbjerg, and G.H. Montgomery, *Cancer-related fatigue: a systematic and meta-analytic review of non-pharmacological therapies for cancer patients.* Psychological bulletin, 2008. **134**(5): p. 700.

32. Beck, A.T., *Cognitive therapy of depression*. 1979: Guilford press.

33. Beck, J.S., *Cognitive behavior therapy: Basics and beyond*. 2011: Guilford Press.

34. Weatherhead, S. and G. Flaherty-Jones, *The Pocket Guide to Therapy: A'how To'of the Core Models*. 2011: Sage.

35. Chalder, T., et al., *Development of a fatigue scale.* Journal of Psychosomatic Research, 1993. **37**(2): p. 147-153.

36. White, P.D., et al., *Comparison of adaptive pacing therapy, cognitive behaviour therapy, graded exercise therapy, and specialist medical care for chronic fatigue syndrome (PACE): a randomised trial.* The Lancet, 2011. **377**(9768): p. 823-836.

37. Johansen, K.L., et al., *Systematic review of the impact of erythropoiesis-stimulating agents on fatigue in dialysis patients.* Nephrology Dialysis Transplantation, 2012. **27**(6): p. 2418-2425.

38. Collister, D., et al., *The effect of erythropoietin-stimulating agents on health-related quality of life in anemia of chronic kidney disease: a systematic review and meta-analysis.* Annals of internal medicine, 2016. **164**(7): p. 472-478.

For peer review only - http://bmjopen.bmj.com/site/about/guidelines.xhtml

39. Dattilio, F.M. and M.A. Hanna, *Collaboration in cognitive□behavioral therapy.* Journal of clinical psychology, 2012. **68**(2): p. 146-158.

40. Horvath, A.O., et al., *Alliance in individual psychotherapy*. 2011, Educational Publishing Foundation.

41. Heins, M.J., H. Knoop, and G. Bleijenberg, *The role of the therapeutic relationship in cognitive behaviour therapy for chronic fatigue syndrome.* Behaviour research and therapy, 2013. **51**(7): p. 368-376.

42. Hudson, J.L., et al., *Improving distress in dialysis (iDiD): a feasibility two-arm parallel randomised controlled trial of an online cognitive behavioural therapy intervention with and without therapist-led telephone support for psychological distress in patients undergoing haemodialysis.* BMJ open, 2016. **6**(4): p. e011286.

43. Hudson, J.L., et al., *Improving Distress in Dialysis (iDiD): A tailored CBT self□management treatment for patients undergoing dialysis.* Journal of Renal Care, 2016. **42**(4): p. 223-238.

44. Moss-Morris, R., et al., *Protocol for the saMS trial (supportive adjustment for multiple sclerosis): a randomized controlled trial comparing cognitive behavioral therapy to supportive listening for adjustment to multiple sclerosis.* BMC neurology, 2009. **9**(1): p. 45.

45. Everitt, H., et al., *Assessing Cognitive behavioural Therapy in Irritable Bowel (ACTIB): protocol for a randomised controlled trial of clinical-effectiveness and cost-effectiveness of therapist delivered cognitive behavioural therapy and web-based self-management in irritable bowel syndrome in adults.* BMJ open, 2015. **5**(7): p. e008622.

For peer review only - http://bmjopen.bmj.com/site/about/guidelines.xhtml

46. Moss-Morris, R., et al., *A pilot randomised controlled trial of an Internet-based cognitive behavioural therapy self-management programme (MS Invigor8) for multiple sclerosis fatigue.* Behaviour Research and Therapy, 2012. **50**(6): p. 415-421.

47. Burgess, M. and T. Chalder, *Overcoming chronic fatigue: A self-help guide using cognitive behavioural techniques.* London: Constable & Robinson Ltd, 2005.

48. Chalder, T., *Coping with chronic fatigue*. 2014: Matrix Audio.

49. Burgess, M. and T. Chalder, *PACE trial: Cognitive Behaviour Therapy for CFS/ME.* 2004: Constable and Robinson.

50. Kazantzis, N., C. Whittington, and F. Dattilio, *Meta□analysis of homework effects in cognitive and behavioral therapy: A replication and extension.* Clinical Psychology: Science and Practice, 2010. **17**(2): p. 144-156.

51. Mausbach, B.T., et al., *The relationship between homework compliance and therapy outcomes: An updated meta-analysis.* Cognitive Therapy and Research, 2010. **34**(5): p. 429-438.

52. Dennison, L. and R. Moss-Morris, *Cognitive–behavioral therapy: what benefits can it offer people with multiple sclerosis?* Expert review of neurotherapeutics, 2010. **10**(9): p. 1383-1390.

53. Roth, A. and S. Pilling, *A competence framework for psychological interventions with people with persistent physical health conditions*. 2015, Retrieved.

54. Eldridge, S.M., et al., *CONSORT 2010 statement: extension to randomised pilot and feasibility trials.* Pilot and feasibility studies, 2016. **2**(1): p. 64.

55. Chilcot, J., et al., *The Chalder Fatigue Questionnaire is a valid and reliable measure of perceived fatigue severity in multiple sclerosis.* Multiple Sclerosis Journal, 2016. **22**(5): p. 677-684.

For peer review only

For peer review only - http://bmjopen.bmj.com/site/about/guidelines.xhtml

56. Picariello, F., et al., *Measuring fatigue in haemodialysis patients: The factor structure of the Chalder Fatigue Questionnaire (CFQ).* Journal of Psychosomatic Research, 2016. **84**: p. 81-83.

57. Chalder, T., J. Tong, and V. Deary, *Family cognitive behaviour therapy for chronic fatigue syndrome: An uncontrolled study.* Archives of Disease in Childhood, 2002. **86**(2): p. 95-97.

58. Mundt, J.C., et al., *The Work and Social Adjustment Scale: A simple measure of impairment in functioning.* The British Journal of Psychiatry, 2002. **180**(5): p. 461-464.

59. Chilcot, J., et al., *Psychosocial and Clinical Correlates of Fatigue in Haemodialysis Patients: the Importance of Patients' Illness Cognitions and Behaviours.* International journal of behavioral medicine, 2016. **23**(3): p. 271-281.

60. Kroenke, K. and R.L. Spitzer, *The PHQ-9: a new depression diagnostic and severity measure.* Psychiatric annals, 2002. **32**(9): p. 509-515.

61. Smarr, K.L. and A.L. Keefer, *Measures of depression and depressive symptoms: Beck Depression Inventory□II (BDI□II), Center for Epidemiologic Studies Depression Scale (CES□D), Geriatric Depression Scale (GDS), Hospital Anxiety and Depression Scale (HADS), and Patient Health Questionnaire□9 (PHQ□9).* Arthritis care & research, 2011. **63**(S11).

62. Kroencke, K., R. Spitzer, and J. Williams, *The PHQ-9: validity of a brief depression severity measure [Electronic version].* Journal of General Internal Medicine, 2001. **16**(9): p. 606-13.

63. Watnick, S., et al., *Validation of 2 depression screening tools in dialysis patients.* American Journal of Kidney Diseases, 2005. **46**(5): p. 919-924.

For peer review only - http://bmjopen.bmj.com/site/about/guidelines.xhtml

64. Spitzer, R.L., et al., *A brief measure for assessing generalized anxiety disorder: the GAD-7.* Archives of internal medicine, 2006. **166**(10): p. 1092-1097.

65. Plummer, F., et al., *Screening for anxiety disorders with the GAD-7 and GAD-2: a systematic review and diagnostic metaanalysis.* General hospital psychiatry, 2016. **39**: p. 24-31.

66. Buysse, D.J., et al., *The Pittsburgh Sleep Quality Index: a new instrument for psychiatric practice and research.* Psychiatry research, 1989. **28**(2): p. 193-213.

67. Carpenter, J.S. and M.A. Andrykowski, *Psychometric evaluation of the Pittsburgh sleep quality index.* Journal of psychosomatic research, 1998. **45**(1): p. 5-13.

68. Burkhalter, H., et al., *Structure validity of the Pittsburgh Sleep Quality Index in renal transplant recipients: A confirmatory factor analysis.* Sleep and Biological Rhythms, 2010. **8**(4): p. 274-281.

69. Broadbent, E., et al., *The brief illness perception questionnaire.* Journal of psychosomatic research, 2006. **60**(6): p. 631-637.

70. Weinman, J., et al., *The illness perception questionnaire: a new method for assessing the cognitive representation of illness.* Psychology and health, 1996. **11**(3): p. 431-445.

71. Moss-Morris, R., et al., *The cognitive and behavioral responses to symptoms questionnaire: measuring cognitive and behavioural aspects of symptom interpretation.* preparation. CIT0034, 2009.

72. Skerrett, T.N. and R. Moss-Morris, *Fatigue and social impairment in multiple sclerosis: the role of patients' cognitive and behavioral responses to their symptoms.* Journal of psychosomatic research, 2006. **61**(5): p. 587-593.

73. Mastin, D.F., J. Bryson, and R. Corwyn, *Assessment of sleep hygiene using the Sleep Hygiene Index.* Journal of behavioral medicine, 2006. **29**(3): p. 223-227.

For peer review only - http://bmjopen.bmj.com/site/about/guidelines.xhtml

74. Cho, S., G.-S. Kim, and J.-H. Lee, *Psychometric evaluation of the sleep hygiene index: a sample of patients with chronic pain.* Health and quality of life outcomes, 2013. **11**(1): p. 213.

75. Booth, M.L., et al., *International physical activity questionnaire: 12-country reliability and validity.* Med sci sports Exerc, 2003. **195**(9131/03): p. 3508-1381.

76. van Poppel, M.N., et al., *Physical activity questionnaires for adults.* Sports medicine, 2010. **40**(7): p. 565-600.

77. Charlson, M.E., et al., *A new method of classifying prognostic comorbidity in longitudinal studies: development and validation.* J Chronic Dis, 1987. **40**(5): p. 373-83.

78. Di Iorio, B., et al., *Charlson Comorbidity Index is a predictor of outcomes in incident hemodialysis patients and correlates with phase angle and hospitalization.* Int J Artif Organs, 2004. **27**(4): p. 330-6.

79. Jassal, S.V., D.E. Schaubel, and S.S.A. Fenton, *Baseline comorbidity in kidney transplant recipients: a comparison of comorbidity indices.* American journal of kidney diseases, 2005. **46**(1): p. 136-142.

80. Moore, G.F., et al., *Process evaluation of complex interventions: Medical Research Council guidance.* bmj, 2015. **350**: p. h1258.

81. Patton, M.Q., *Two decades of developments in qualitative inquiry a personal, experiential perspective.* Qualitative Social Work, 2002. **1**(3): p. 261-283.

82. Guest, G., A. Bunce, and L. Johnson, *How many interviews are enough? An experiment with data saturation and variability.* Field Methods, 2006. **18**(1): p. 59-82.

83. Billingham, S.A., A.L. Whitehead, and S.A. Julious, *An audit of sample sizes for pilot and feasibility trials being undertaken in the United Kingdom registered in the United*

For peer review only - http://bmjopen.bmj.com/site/about/guidelines.xhtml

Kingdom Clinical Research Network database. BMC medical research methodology, 2013. **13**(1): p. 104.

84. Viechtbauer, W., et al., *A simple formula for the calculation of sample size in pilot studies.* Journal of clinical epidemiology, 2015. **68**(11): p. 1375-1379.

85. Browne, R.H., *On the use of a pilot sample for sample size determination.* Statistics in medicine, 1995. **14**(17): p. 1933-1940.

86. Tavakol, M. and R. Dennick, *Making sense of Cronbach's alpha.* International journal of medical education, 2011. **2**: p. 53.

87. Braun, V., V. Clarke, and G. Terry, *Thematic analysis.* Qual Res Clin Health Psychol, 2014: p. 95-114.

88. Senn, B., et al., *Developing and evaluating complex interventions: the new Medical Research Council guidance.* Studies, 2013. **59**: p. 587-592.

Figure 1. CBT model of renal fatigue. This diagram illustrates the different clinical, social-situational, and psychological factors that contribute to fatigue in this setting.

Figure 2. Anticipated flow of participants through the study. Number of patients approached for screen, those who consented, and those who were assessed for eligibility will be recorded. Eligible patients will be invited to complete a baseline questionnaire (T0). After completion of the baseline questionnaire, participants will be randomised. Participants in the intervention arm will receive the intervention over 4 to 6 weeks. All participants will complete a follow-up questionnaire at 3 months post-randomisation (T1). Participants in the intervention arm will be invited to take part in a qualitative evaluation interview at the end of their involvement in the study. After completion of the follow-up questionnaire, participants in the control arm will receive the intervention materials.

Figure 3. Structure and content of the intervention. The intervention will follow a stepped approach over 4 to 6 weeks, accompanied by 3 to 5 sessions with a therapist. In Level 1, participants will cover chapters 1, 2, a relevant chapter identified in the assessment, and chapter 8. Level 2 focuses on cognitive therapy.

For peer review only

For peer review only - http://bmjopen.bmj.com/site/about/guidelines.xhtml

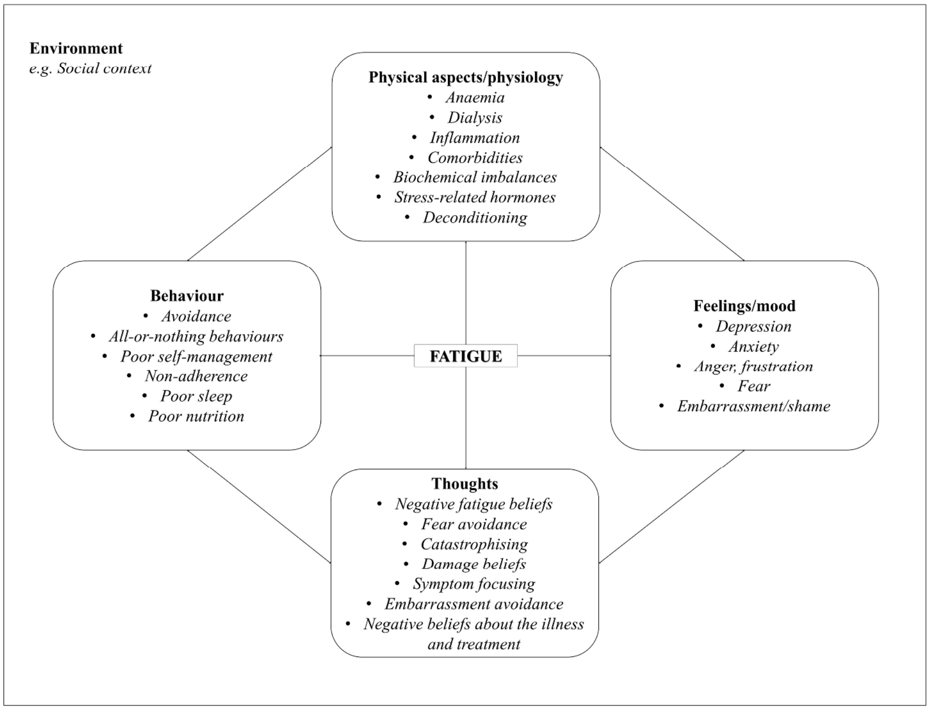

Figure 1. CBT model of renal fatigue.

272x272mm (300 x 300 DPI)

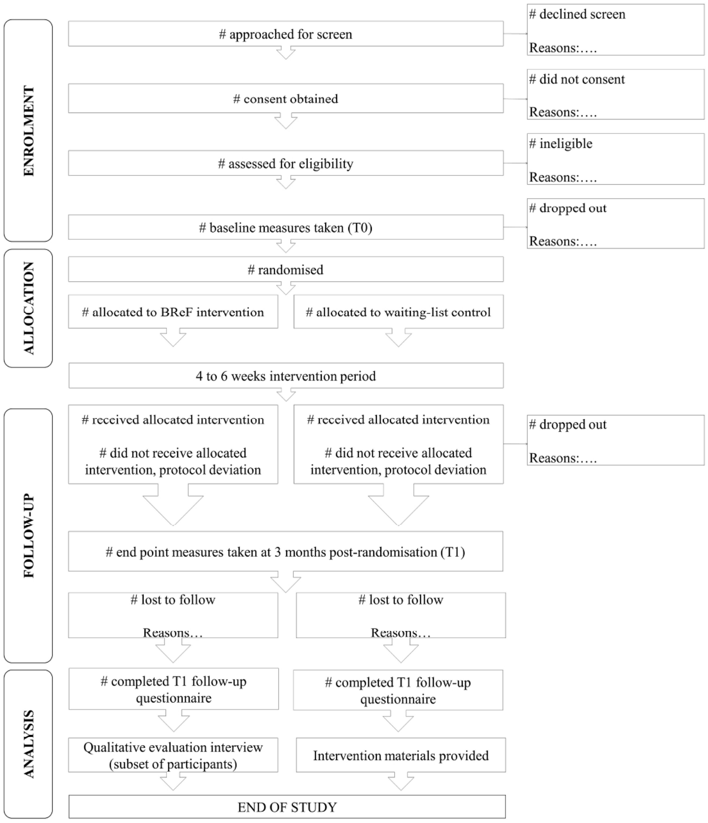

Figure 2. Anticipated flow of participants through the study.

230x194mm (300 x 300 DPI)

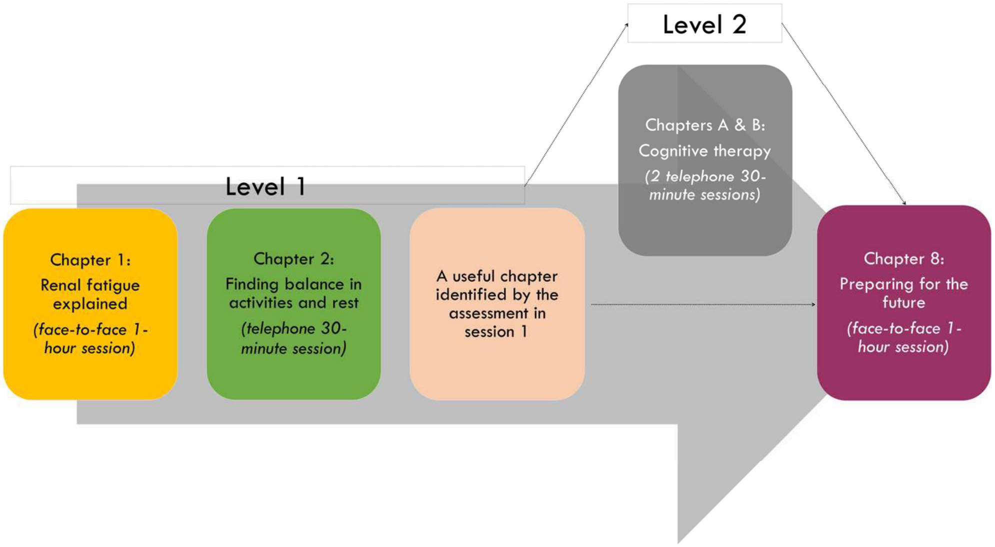

Figure 3. Structure and content of the intervention.

146x82mm (300 x 300 DPI)

STANDARD PROTOCOL ITEMS: RECOMMENDATIONS FOR INTERVENTIONAL TRIALS

SPIRIT 2013 Checklist: Recommended items to address in a clinical trial protocol and related documents*

Section/item	Item No	Description	Addressed on page number
**Administrative information**			
Title	1	Descriptive title identifying the study design, population, interventions, and, if applicable, trial acronym	page 1
Trial registration	2a	Trial identifier and registry name. If not yet registered, name of intended registry	page 1 (ISRCTN91238019)
	2b	All items from the World Health Organization Trial Registration Data Set	ISRCTN registration form
Protocol version	3	Date and version identifier	page 1
Funding	4	Sources and types of financial, material, and other support	pages 23-24
Roles and responsibilities	5a	Names, affiliations, and roles of protocol contributors	page 1
	5b	Name and contact information for the trial sponsor	ISRCTN registration form
	5c	Role of study sponsor and funders, if any, in study design; collection, management, analysis, and interpretation of data; writing of the report; and the decision to submit the report for publication, including whether they will have ultimate authority over any of these activities	page 23

For peer review only - http://bmjopen.bmj.com/site/about/guidelines.xhtml

	5d	Composition, roles, and responsibilities of the coordinating centre, steering committee, endpoint adjudication committee, data management team, and other individuals or groups overseeing the trial, if applicable (see Item 21a for data monitoring committee)	N/A small feasibility trial
**Introduction**			
Background and rationale	6a	Description of research question and justification for undertaking the trial, including summary of relevant studies (published and unpublished) examining benefits and harms for each intervention	pages 4-5
	6b	Explanation for choice of comparators	page 14
Objectives	7	Specific objectives or hypotheses	pages-5-6
Trial design	8	Description of trial design including type of trial (eg, parallel group, crossover, factorial, single group), allocation ratio, and framework (eg, superiority, equivalence, noninferiority, exploratory)	page 6
**Methods: Participants, interventions, and outcomes**			
Study setting	9	Description of study settings (eg, community clinic, academic hospital) and list of countries where data will be collected. Reference to where list of study sites can be obtained	page 6
Eligibility criteria	10	Inclusion and exclusion criteria for participants. If applicable, eligibility criteria for study centres and individuals who will perform the interventions (eg, surgeons, psychotherapists)	pages 6-7
Interventions	11a	Interventions for each group with sufficient detail to allow replication, including how and when they will be administered	pages 8-13
	11b	Criteria for discontinuing or modifying allocated interventions for a given trial participant (eg, drug dose change in response to harms, participant request, or improving/worsening disease)	N/A
	11c	Strategies to improve adherence to intervention protocols, and any procedures for monitoring adherence (eg, drug tablet return, laboratory tests)	page 15
	11d	Relevant concomitant care and interventions that are permitted or prohibited during the trial	N/A

For peer review only - http://bmjopen.bmj.com/site/about/guidelines.xhtml

Outcomes	12	Primary, secondary, and other outcomes, including the specific measurement variable (eg, systolic blood pressure), analysis metric (eg, change from baseline, final value, time to event), method of aggregation (eg, median, proportion), and time point for each outcome. Explanation of the clinical relevance of chosen efficacy and harm outcomes is strongly recommended	pages 15-19
Participant timeline	13	Time schedule of enrolment, interventions (including any run-ins and washouts), assessments, and visits for participants. A schematic diagram is highly recommended (see Figure)	pages 7-8, Figure 2 and Table 3.
Sample size	14	Estimated number of participants needed to achieve study objectives and how it was determined, including clinical and statistical assumptions supporting any sample size calculations	page 20
Recruitment	15	Strategies for achieving adequate participant enrolment to reach target sample size	pages 9 and 14
**Methods: Assignment of interventions (for controlled trials)**			
Allocation:			
Sequence generation	16a	Method of generating the allocation sequence (eg, computer-generated random numbers), and list of any factors for stratification. To reduce predictability of a random sequence, details of any planned restriction (eg, blocking) should be provided in a separate document that is unavailable to those who enrol participants or assign interventions	page 8
Allocation concealment mechanism	16b	Mechanism of implementing the allocation sequence (eg, central telephone; sequentially numbered, opaque, sealed envelopes), describing any steps to conceal the sequence until interventions are assigned	page 8
Implementation	16c	Who will generate the allocation sequence, who will enrol participants, and who will assign participants to interventions	page 8
Blinding (masking)	17a	Who will be blinded after assignment to interventions (eg, trial participants, care providers, outcome assessors, data analysts), and how	page 8
	17b	If blinded, circumstances under which unblinding is permissible, and procedure for revealing a participant's allocated intervention during the trial	page 8
**Methods: Data collection, management, and analysis**			

For peer review only - http://bmjopen.bmj.com/site/about/guidelines.xhtml

Data collection methods	18a	Plans for assessment and collection of outcome, baseline, and other trial data, including any related processes to promote data quality (eg, duplicate measurements, training of assessors) and a description of study instruments (eg, questionnaires, laboratory tests) along with their reliability and validity, if known. Reference to where data collection forms can be found, if not in the protocol	pages 14-17
	18b	Plans to promote participant retention and complete follow-up, including list of any outcome data to be collected for participants who discontinue or deviate from intervention protocols	N/A feasibility trial
Data management	19	Plans for data entry, coding, security, and storage, including any related processes to promote data quality (eg, double data entry; range checks for data values). Reference to where details of data management procedures can be found, if not in the protocol	N/A small feasibility trial
Statistical methods	20a	Statistical methods for analysing primary and secondary outcomes. Reference to where other details of the statistical analysis plan can be found, if not in the protocol	pages 20-22
	20b	Methods for any additional analyses (eg, subgroup and adjusted analyses)	pages 20-22
	20c	Definition of analysis population relating to protocol non-adherence (eg, as randomised analysis), and any statistical methods to handle missing data (eg, multiple imputation)	Feasibility trial, only exploratory analysis
**Methods: Monitoring**			
Data monitoring	21a	Composition of data monitoring committee (DMC); summary of its role and reporting structure; statement of whether it is independent from the sponsor and competing interests; and reference to where further details about its charter can be found, if not in the protocol. Alternatively, an explanation of why a DMC is not needed	No formal DMC, as it is a small feasibility trial, but monthly steering meetings (page 23)
	21b	Description of any interim analyses and stopping guidelines, including who will have access to these interim results and make the final decision to terminate the trial	pages 14 (clinical supervision), 17-18, 23

For peer review only - http://bmjopen.bmj.com/site/about/guidelines.xhtml

Harms	22	Plans for collecting, assessing, reporting, and managing solicited and spontaneously reported adverse events and other unintended effects of trial interventions or trial conduct	pages 14 and 18
Auditing	23	Frequency and procedures for auditing trial conduct, if any, and whether the process will be independent from investigators and the sponsor	pages 22-23
**Ethics and dissemination**			
Research ethics approval	24	Plans for seeking research ethics committee/institutional review board (REC/IRB) approval	pages 22-23
Protocol amendments	25	Plans for communicating important protocol modifications (eg, changes to eligibility criteria, outcomes, analyses) to relevant parties (eg, investigators, REC/IRBs, trial participants, trial registries, journals, regulators)	pages 22-23
Consent or assent	26a	Who will obtain informed consent or assent from potential trial participants or authorised surrogates, and how (see Item 32)	Pages 7, 22
	26b	Additional consent provisions for collection and use of participant data and biological specimens in ancillary studies, if applicable	N/A
Confidentiality	27	How personal information about potential and enrolled participants will be collected, shared, and maintained in order to protect confidentiality before, during, and after the trial	pages 22-23
Declaration of interests	28	Financial and other competing interests for principal investigators for the overall trial and each study site	page 24
Access to data	29	Statement of who will have access to the final trial dataset, and disclosure of contractual agreements that limit such access for investigators	page 23 (small feasibility trial)
Ancillary and post-trial care	30	Provisions, if any, for ancillary and post-trial care, and for compensation to those who suffer harm from trial participation	page 23
Dissemination policy	31a	Plans for investigators and sponsor to communicate trial results to participants, healthcare professionals, the public, and other relevant groups (eg, via publication, reporting in results databases, or other data sharing arrangements), including any publication restrictions	page 23
	31b	Authorship eligibility guidelines and any intended use of professional writers	page 23

For peer review only - http://bmjopen.bmj.com/site/about/guidelines.xhtml

	31c	Plans, if any, for granting public access to the full protocol, participant-level dataset, and statistical code	ISRCTN registration: The data sharing plans for the current study are unknown and will be made available at a later date.
**Appendices**			
Informed consent materials	32	Model consent form and other related documentation given to participants and authorised surrogates	Available on request
Biological specimens	33	Plans for collection, laboratory evaluation, and storage of biological specimens for genetic or molecular analysis in the current trial and for future use in ancillary studies, if applicable	N/A

*It is strongly recommended that this checklist be read in conjunction with the SPIRIT 2013 Explanation & Elaboration for important clarification on the items. Amendments to the protocol should be tracked and dated. The SPIRIT checklist is copyrighted by the SPIRIT Group under the Creative Commons "Attribution-NonCommercial-NoDerivs 3.0 Unported" license.

For peer review only

For peer review only - http://bmjopen.bmj.com/site/about/guidelines.xhtml
